# Nationwide Trends in Newborn Anthropometric Indicators in Kazakhstan, 2013–2022: A Population-Level Time-Trend Analysis

**DOI:** 10.3390/children13081069

**Published:** 2026-08-12

**Authors:** Marat Shoranov, Anel Ibrayeva, Yerlan Ismoldayev, Bolat Sadykov, Dimash Davletov, Shynar Tanabayeva, Ildar Fakhradiyev

**Affiliations:** 1 Department of Medicine, S.D. Asfendiyarov Kazakh National Medical University, Almaty 050000, Kazakhstandavletov.d@kaznmu.kz (D.D.);; 2 College of Medicine, Korea University, Seoul 02841, Republic of Korea

**Keywords:** newborn anthropometry, birth weight, birth length, low birth weight, macrosomia, Rohrer index, Kazakhstan

## Abstract

**Highlights:**

**What are the main findings?**

• Kazakhstan newborns grew larger from 2013 to 2022, while low birth weight fell and macrosomia rose.
• Regional newborn trends varied significantly across Kazakhstan’s territories.

**What arethe implications of the main findings?**

• Both birth-weight extremes need simultaneous, ongoing population surveillance.
• Regional surveillance should account for referral patterns and registry completeness.

**Abstract:**

**Background/Objectives:** Birth size is a population-level marker of fetal growth, maternal health, and perinatal care. In Kazakhstan, long-term national and regional patterns in newborn anthropometry have not been jointly examined across birth weight, birth length, the Rohrer index, low birth weight (LBW), and macrosomia. This study aimed to quantify national annual trends in these five indicators for 2013–2022, compare region-specific trajectories, and examine whether the two ends of the birth-weight distribution changed in different directions. **Methods:** This retrospective ecological time-trend study used de-identified individual newborn records (birth weight, birth length, sex, birth date, and region; 3,483,810 live births, 2013–2022) from the Electronic Register of Inpatients of the Ministry of Health of the Republic of Kazakhstan. These individual-level records were used to calculate the Rohrer index and to apply exclusion criteria at the newborn level, but national trend models were deliberately fitted on the 10 annual observations, and regional heterogeneity was tested with a single region × year interaction model rather than individual births or independent per-region regressions to avoid pseudoreplication. **Results:** Mean birth weight increased from 3429.5 g (2013) to 3518.4 g (2022), and mean birth length increased from 52.5 to 53.3 cm. Recorded LBW prevalence declined from 3.09% to 1.46%, while macrosomia increased from 12.59% to 16.02%. Pooled regional birth weight ranged from 3392.6 g to 3548.6 g, LBW from 0.9% to 5.3%, and macrosomia from 11.7% to 18.1%; region-specific trends after false-discovery-rate correction showed that only North Kazakhstan Region had a statistically robust increase in LBW. **Conclusions:** Official registry data for Kazakhstan, analyzed at the individual level and aggregated for trend modeling, showed a decade-long increase in mean birth weight and length, an observed decline in recorded LBW prevalence, and an increase in macrosomia prevalence, with statistically confirmed heterogeneity in regional trajectories. Because this study lacked gestational age or maternal characteristics, these findings should be interpreted as ecological surveillance patterns that support continued monitoring of both ends of the birth-weight distribution, rather than as evidence of individual-level changes in fetal growth or maternal metabolic risk.

## 1. Introduction

Birth size, particularly birth weight and birth length, is widely used as an accessible population-level indicator of fetal growth, intrauterine conditions, and perinatal health [1]. Routinely collected birthweight data can support surveillance of newborn vulnerability, monitoring of inequalities in birth outcomes, and detection of shifts in population risk profiles, provided that registration and measurement practices are sufficiently complete, accurate, and consistent over time [2,3]. At the individual level, birth weight and length are additionally shaped by gestational age, fetal sex [4], placental function [5], maternal anthropometry and metabolic status [6], parity, smoking and antenatal care [7], and intrapartum-care quality [8].

Newborn anthropometry is best characterized through several complementary indicators rather than mean birth weight alone. Low birth weight (LBW, birth weight < 2500 g) remains one of the most widely used maternal-and-child-health surveillance indicators. Global estimates suggest approximately 19.8 million newborns (14.7% of live births) had LBW in 2020, with progress toward global reduction targets slower than required [9,10]. LBW is a composite indicator that can result from preterm birth, fetal growth restriction, or both, and population trends in LBW cannot be interpreted mechanistically without gestational age and fetal growth data [9,11,12]. At the opposite end of the distribution, macrosomia (commonly defined using an absolute threshold of 4000 g or 4500 g) is associated with a higher probability of labor complications, shoulder dystocia, operative delivery, and neonatal metabolic complications, and has been linked in the literature with maternal obesity, diabetes, excessive gestational weight gain, and multiparity [13,14,15,16]. Birth length and the Rohrer (ponderal) index were included in this study to examine whether temporal changes in birth weight were accompanied by parallel changes in newborn length and in population-level weight-for-length proportionality. Because only aggregated means are available, this proportionality question is necessarily addressed at the population level and cannot describe individual newborn body composition [17,18].

Together, monitoring LBW and macrosomia in parallel is informative because population-based studies have shown that the lower and upper tails of the birth-weight distribution can move in different directions over time. For example, studies from China have reported heterogeneous trends in LBW, macrosomia, and gestational-age-specific birth-size indicators related to maternal age, parity, diabetes, pre-pregnancy BMI, and obstetric practice [19]. Three recent Kazakhstan-based studies provide relevant but partial evidence: an analysis of the association between birth weight and infant mortality [20], a customized birthweight standard for the Kazakhstani population [21], and an analysis of socioeconomic and demographic correlates of infant mortality [22]. None of these studies jointly examined decade-long national and region-specific trends in birth weight, birth length, the Rohrer index, LBW, and macrosomia together; this combination is the specific gap addressed here.

Kazakhstan is a relevant setting for this surveillance question because harmonized national and regional statistical series spanning a full decade are publicly available, which is a precondition for describing secular trends. Documenting these trends is relevant because it can identify which regions and which end of the birth-weight distribution may require closer surveillance attention, informing where maternal-metabolic-risk or fetal-growth-restriction-focused monitoring should be prioritized. The study period, from 2013 to 2022, was chosen because it is the longest span for which a territorially harmonized annual series was available in the source tables. The series was not extended past 2022 because the 2022 territorial reorganization (creation of Abai, Zhetysu and Ulytau regions) limits comparability of subsequent years within the same harmonized categories.

Against this background, the present study had three specific objectives: (a) to quantify national annual trends in mean birth weight, mean birth length, the Rohrer index, LBW, and macrosomia in Kazakhstan during 2013–2022; (b) to estimate and formally compare region-specific trajectories for these outcomes; and (c) to characterize simultaneous changes in the prevalence of LBW and macrosomia, i.e., whether the two ends of the birth-weight distribution changed in different directions.

## 2. Materials and Methods

### 2.1. Study Design and Data Sources

This was a nationwide retrospective ecological time-trend study based on de-identified individual-level registry records for Kazakhstan, which were subsequently aggregated into national-year and region-year observations for trend modeling. Data were obtained from the public dataset “Data on newborns in the Republic of Kazakhstan without personal data”, published on the Open Data Portal of the Republic of Kazakhstan (ashyq.data.gov.kz), sourced from the Electronic Register of Inpatients of the Ministry of Health of the Republic of Kazakhstan (ERSB MH RK). The merged dataset and details on the process of data acquisition can be found in DOI (https://doi.org/10.5281/zenodo.21617508) [23].

The dataset consists of de-identified individual newborn records and contains no names, national identifiers, addresses, or other direct or indirect personal identifiers. Hence, birth weight, birth length, sex, birth date, and region are the only fields provided. A supplementary data dictionary (Supplementary Table S7) maps each of the five extracted fields (birth weight, birth length, sex, birth date, and region) to its corresponding manuscript variable, unit of measurement, available years, and geographic level, and states whether each manuscript indicator (e.g., the Rohrer index, LBW, and macrosomia) was extracted directly or subsequently calculated by the authors.

Because the register captures inpatient (in-facility) deliveries only, the analytical population is not a complete census of live births in Kazakhstan. To quantify ascertainment, annual eligible record counts were compared with the corresponding official national live-birth totals published by the Bureau of National Statistics of the Agency for Strategic Planning and Reforms of the Republic of Kazakhstan (BNS), an independent vital-statistics source. Births occurring outside medical facilities, and any in-facility births not entered into the register, are therefore not represented, and the extent of non-ascertainment increased over the study period. The BNS data and details were also deposited in the DOI (https://doi.org/10.5281/zenodo.21617508).

### 2.2. Study Population, Eligibility, and Denominators

The analysis included all live-birth records present in the source file for 2013–2022. The source file contained 3,487,406 live-birth records for this window before any exclusion. The reported total of 3,483,810 therefore represents records remaining after all exclusions shown in Supplementary Table S9. The source registry is described by the Ministry of Health as covering inpatient birth registrations nationally. For birth weight, birth length, and the Rohrer index, the analytical denominator was the number of newborns with a valid recorded value for that field after the exclusions. For LBW and macrosomia, the denominator was the total number of births with a valid weight record. Because the source file does not separately flag how many births lacked a valid anthropometric measurement, the LBW and macrosomia prevalences reported here should be read as prevalence among newborns with a recorded weight, which may not equal prevalence among all live births if measurement was incomplete for some subgroup of births.

### 2.3. Study Period and Territorial Harmonization

Administrative-territorial changes during 2013–2022 were handled as follows, and they are reproduced in full, year by year, in Supplementary Table S8. Records labeled “г. Астана (действoвавший дo 23 March 2019)” (Astana City, pre-23 March 2019 designation) were merged into the single category “Astana City” to preserve a continuous series across the 2019 renaming. Shymkent City, which separated administratively from South Kazakhstan Region in 2018, was retained as a distinct category across the full 2013–2022 series, as it was already coded separately in the source file for all study years; South Kazakhstan Region records were merged into “Turkistan Region”, the successor designation, to preserve continuity. Both Shymkent City and Turkistan Region therefore have complete, non-zero record counts in every one of the 10 study years (Supplementary Table S8), so no further adjustment was required for these two territories. The three regions created in 2022 (Abai, Zhetysu, and Ulytau) were not analyzed as separate categories: in this data extract, records nominally attributable to Zhetysu and Ulytau appear only from 2023 onward (outside the study window), and no separate Abai code appears at all, so all three 2022 successor regions are implicitly retained within their pre-2022 parent regions (East Kazakhstan Region for Abai; Almaty Region for Zhetysu; Karaganda Region for Ulytau) throughout 2013–2022.

### 2.4. Variables and Operational Definitions

The extracted indicators were birth weight (g), birth length (cm), sex, birth date, and region, recorded individually for every newborn. From these, calendar year was derived from birth date. LBW was defined as birth weight < 2500 g, macrosomia was defined as birth weight ≥ 4000 g, and the Rohrer index was calculated for every newborn individually as (birth weight in g/birth length in cm^3^) × 100, before any averaging. Biologically implausible records (birth weight < 500 g or >10,000 g; birth length < 25 cm or >70 cm) and records with an unrecognized sex code were excluded at the individual-record level. The 500 g lower threshold follows the WHO’s live-birth registration convention, under which a recorded weight below this level is considered more likely to reflect a measurement or data-entry error than a viable, correctly recorded live birth, in a registry that does not itself record gestational age and so cannot otherwise distinguish an extremely preterm live birth from a data error. Supplementary Table S9 reports the number of records excluded by each criterion, by year, so that the final analytical N in every national-year and region-year cell can be independently reconciled against the raw source file.

### 2.5. Rohrer Index: Calculation and Provenance

The Rohrer (ponderal) index was not reported directly in the source dataset, and it was calculated by the authors for every individual newborn as (weight [g]/length [cm]^3^) × 100, and only then averaged within each year or region. This ordering matters: recalculating a “mean” Rohrer index from already-aggregated mean weight and mean length instead (mean weight/mean length^3^ × 100) is not equivalent to the mean of individual indices and would introduce a small but non-zero bias. All Rohrer index results reported in this manuscript use the mean-of-individual-indices method throughout.

### 2.6. Statistical Analysis

No imputation, interpolation, or back-calculation was performed for unavailable data. Where a value was not available in the source tables, it is indicated with an em dash (—), and the corresponding cell is excluded from the relevant denominator (Supplementary Table S9). All analyses were conducted in Python 3.10, using pandas (1.5) for data handling, statsmodels (0.14) for regression modeling (statsmodels.api.WLS and statsmodels.api.GLSAR for the continuous national and sensitivity models; statsmodels.api.GLM with family = Binomial() for the binary national and regional models, fit with scale set to the estimated Pearson dispersion for the quasi-binomial specification; and statsmodels.stats.multitest.multipletests for Benjamini–Hochberg FDR correction), and scipy (1.11) for auxiliary statistical functions. Because the analysis used the individual-level extract described in Section 2.1, aggregated by the authors, it did not separately estimate individual-level multivariable associations, sex-adjusted anthropometric effects, or individual-level prediction models—those would require covariates (gestational age and maternal characteristics) that are not present in the source fields at all, independent of the individual-versus-aggregate question. The de-identified individual-level analytical extract, the cleaned national-year and region-year aggregate datasets, the data-source URLs (with accessed dates), and the complete analysis code were deposited at the Kazakh National Medical University Research Repository on Zenodo, https://doi.org/10.5281/zenodo.21617508 (v3, published 28 July 2026).

#### 2.6.1. National Trend Analysis

For the three continuous national outcomes (mean birth weight, mean birth length, and Rohrer index), the unit of analysis was the annual observation (n = 10, 2013–2022), not the individual birth. Calendar year was centered at 2013 (year_c = calendar year − 2013) and modeled as a continuous predictor. The primary model was weighted least squares (WLS) of the 10 annual means on year, with each year weighted by its number of births. This reflects that years with more births are measured more precisely while avoiding the pseudoreplication that would result from using individual births as the unit of statistical precision for a variable, calendar year, that in fact varies across only 10 values. The 95% confidence intervals (95% CI) and exact *p*-values for the annual slope were obtained from this WLS model. The model-estimated total change over the study period was calculated as the slope multiplied by 9 (years), with its 95% CI obtained by the corresponding linear combination of the model’s parameter covariance matrix.

For the two binary national outcomes (LBW and macrosomia), the response in each annual observation was the number of affected infants out of the total live births with a valid weight record for that year (a grouped binomial response, not an individual-level binary outcome). A generalized linear model (GLM) with binomial family and logit link was fitted to these 10 annual observations: logit(p_year) = β_0_ + β_1_ · year_c, where year_c is calendar year centered at 2013, and p_year is the annual proportion of affected infants. The odds ratio (OR) for a one-year increase in calendar year is exp(β_1_), with its 95% CI obtained from the Wald confidence interval for β_1_ on the log-odds scale and back-transformed by exponentiation.

Overdispersion was formally assessed for each outcome as the ratio of the Pearson chi-square statistic to its residual degrees of freedom from the standard binomial fit. Because this ratio substantially exceeded 1 for both LBW and macrosomia, the primary model reported is quasi-binomial: the same point estimates (β_0_, β_1_, and hence the OR) as the standard binomial GLM, but with standard errors, confidence intervals, and *p*-values scaled by the square root of the estimated dispersion parameter, which widens all three relative to the (invalid) standard binomial assumption without changing the odds ratio itself. Predicted prevalence for 2013 and 2022 was obtained by back-transforming the fitted linear predictor at year_c = 0 and year_c = 9, respectively, with 95% CIs from the delta method applied to the linear predictor. The annual absolute change in percentage points was likewise obtained by the delta method, as the derivative of predicted prevalence with respect to year_c evaluated at the mean study year (2017.5), together with its delta-method standard error and 95% CI. Temporal autocorrelation of the annual residuals from the binomial fit was assessed alongside the continuous-outcome diagnostics.

For the regional analysis of LBW and macrosomia, the same grouped-binomial/quasi-binomial specification was used, replacing the single national year_c predictor with region, year, and, for the unified interaction test, a region × year interaction term. Because the quasi-binomial model does not have a classical likelihood-ratio chi-square, the interaction was tested by comparing the quasi-binomial deviances of the interaction and main-effects-only models via an F-test (as for the continuous outcomes), which is the standard approach for testing nested terms under an estimated dispersion parameter.

#### 2.6.2. Regional Trend Analysis

Region-specific trend estimates were obtained by fitting the same WLS (continuous outcomes) or quasi-binomial (LBW and macrosomia) models independently within each of the 17 harmonized regions (each with its own 10 annual region-year observations). All 17 regions had non-missing data for every one of the 10 years, so no region-year cell required special handling for missing years. Region-specific regressions were conducted for four outcomes—birth weight, birth length, LBW, and macrosomia—rather than for all five outcomes examined elsewhere in this study. The Rohrer index was intentionally not included in this per-region family because it is a deterministic function of birth weight and birth length, both of which were already tested per region. Adding a third, non-independent per-region test would not contribute new information while also diluting the false-discovery-rate correction applied across the family. Because 17 independent regressions were therefore fitted for each of these four outcomes (68 tests in total), Benjamini–Hochberg false-discovery-rate (FDR) correction was applied jointly across all 68 region-level *p*-values together (i.e., across all four outcomes and all 17 regions as a single family), not separately within each outcome, and both raw and FDR-adjusted *p*-values are reported. Critically, these per-region regressions describe region-specific trajectories but do not by themselves constitute a formal test of whether the pace of change differs across territories. That formal test was instead obtained from a single unified model containing region, year, and a region × year interaction term (WLS for continuous outcomes; quasi-binomial for LBW/macrosomia), fitted on the 170 region-year observations (weighted by region-year live-birth counts), comparing the fit of this interaction model against a reduced model with region and year main effects only via an F-test. This keeps the model well short of saturation (136 residual degrees of freedom) so that the interaction can be tested formally, and this test is the basis for any statement that regional trends differed significantly.

### 2.7. Model Diagnostics and Sensitivity Analyses

For every national continuous-outcome model, we examined plots of observed versus model-fitted annual values, residual-versus-year plots, the Durbin–Watson statistic for residual autocorrelation, and Cook’s distance to identify influential years. We additionally compared the linear specification against a quadratic-in-year specification via an F-test to formally assess non-linearity, and we fitted a GLSAR(1) model that estimates and adjusts for first-order autocorrelation between consecutive annual means. Because the final years of the series (2020–2022) coincide with the COVID-19 pandemic, we performed a pre-specified sensitivity analysis refitting every national model restricted to 2013–2019. Because sex is available in the source data, we additionally fitted sex-stratified national trend models for all five outcomes as a sensitivity analysis. Finally, because territorial harmonization required judgment calls for the 2022 administrative reorganization, we repeated the unified region × year interaction model after excluding the three regions affected by a 2022 split (East Kazakhstan Region, Almaty Region, and Karaganda Region) to assess whether the main regional conclusions were sensitive to this choice.

## 3. Results

### 3.1. National Descriptive Trend

**Figure 1:**
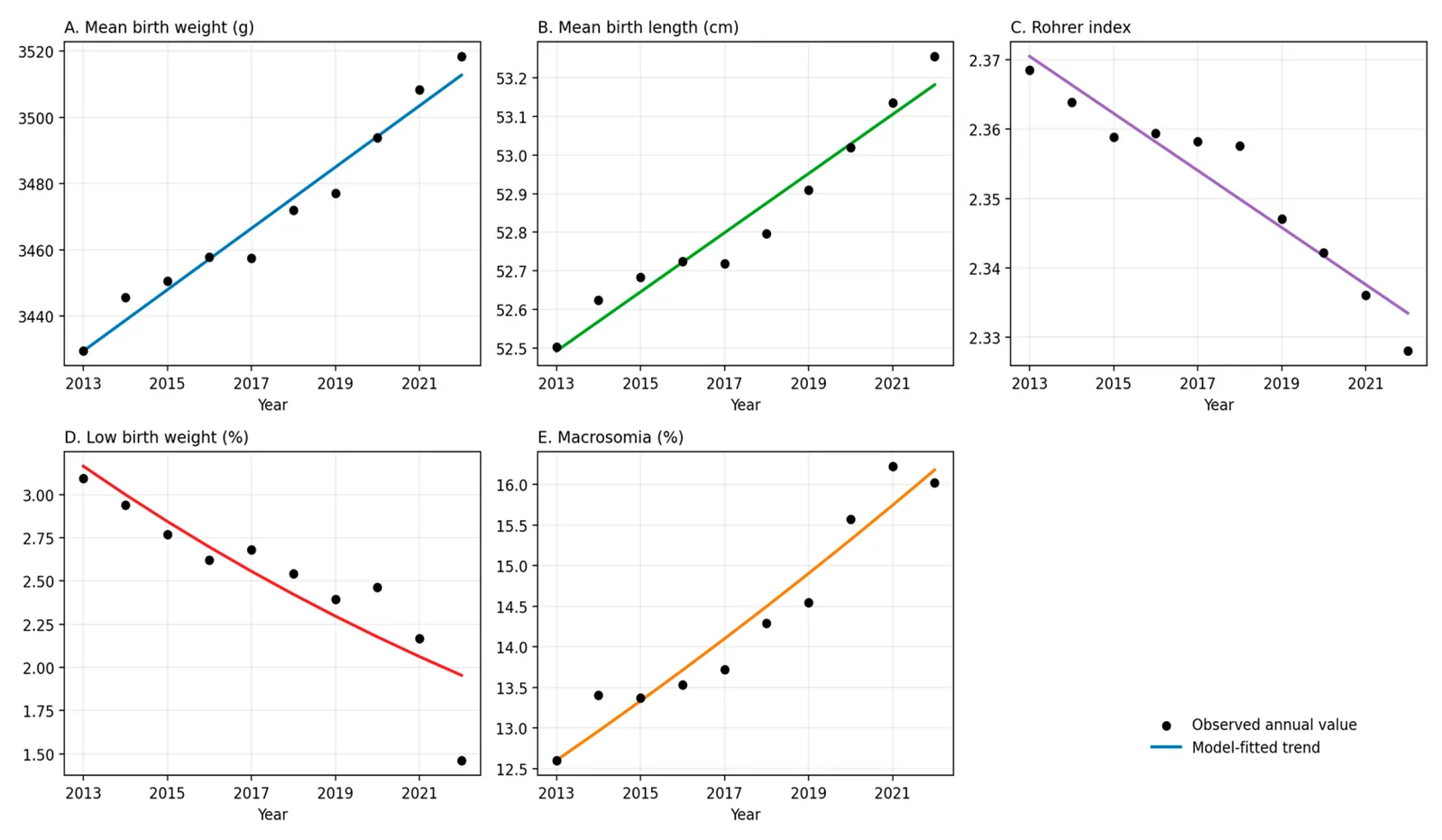
National trends in newborn anthropometric indicators, Kazakhstan, 2013–2022. (**A**) Mean birth weight, (**B**) mean birth length, (**C**) Rohrer index, (**D**) low birth weight, and (**E**) macrosomia. Points show observed annual values, and lines show the model-fitted trend from the primary national model.

A total of 3,483,810 live births during 2013–2022 were included (Table 1). The proportion of male births ranged from 51.0% to 51.5% across the study period. Mean birth weight increased from 3429.5 g (2013) to 3518.4 g (2022); mean birth length increased from 52.5 cm to 53.3 cm; and the Rohrer index decreased from 2.369 to 2.328. Recorded LBW prevalence decreased from 3.09% to 1.46%; and recorded macrosomia prevalence increased from 12.59% to 16.02%. These changes correspond to a descriptive difference, calculated directly from the 2013 and 2022 annual values in Table 1, of +88.9 g for birth weight, +0.8 cm for birth length, −1.63 percentage points for LBW, and +3.43 percentage points for macrosomia. Compared with independent BNS regional live-birth totals, these records represented 93.3% of officially registered live births over the study period, with annual coverage declining from 95.1% in 2013 to 87.7% in 2022 (Supplementary Table S13). These national series are shown in Figure 1.

### 3.2. Pooled Regional Indicators

**Figure 2:**
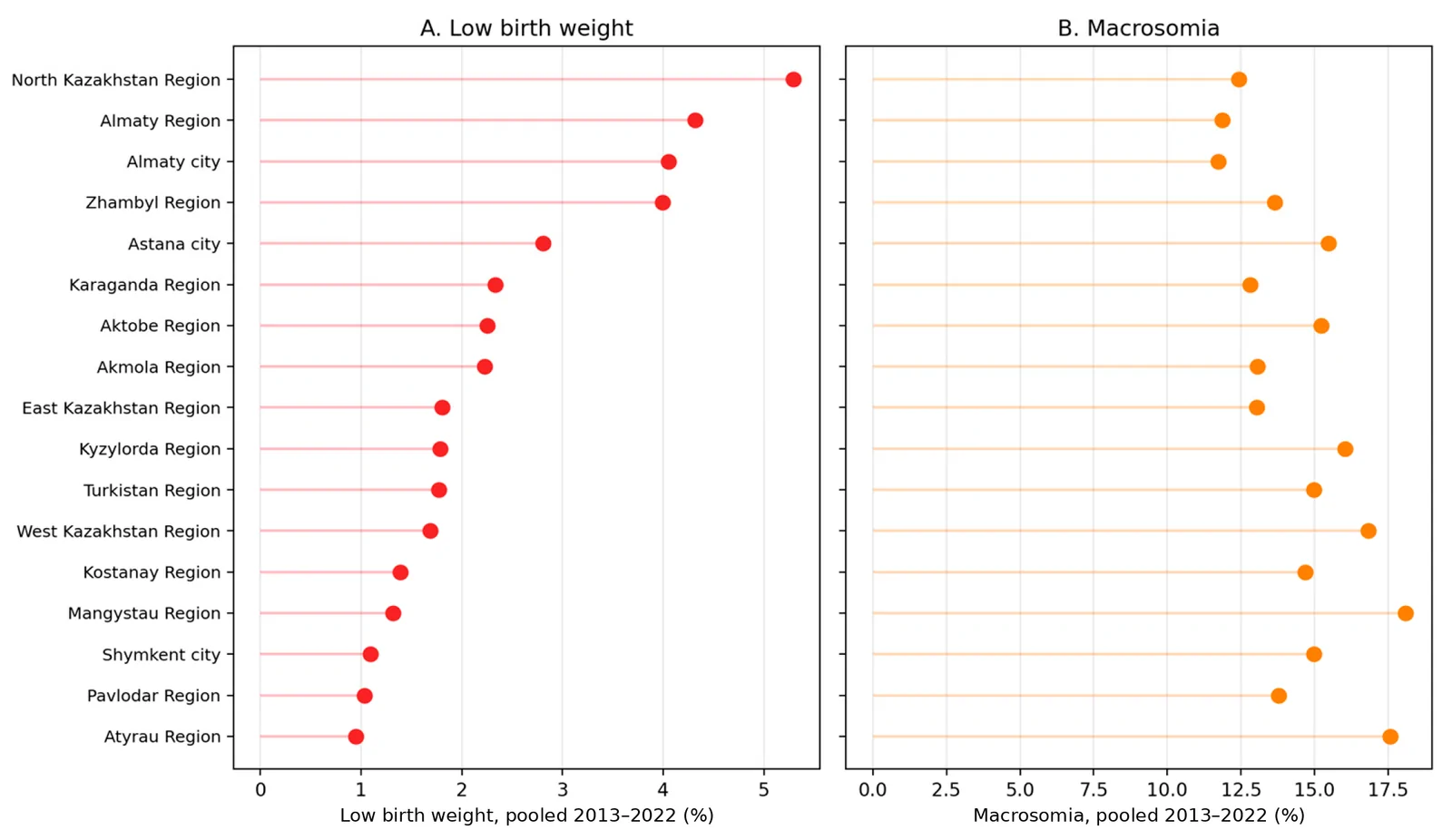
Regional variation in low birth weight and macrosomia, Kazakhstan, 2013–2022. Two aligned horizontal dot plots with separate x-axis scales for (**A**) low birth weight and (**B**) macrosomia; regions are ordered by decreasing pooled low-birth-weight prevalence in both panels to allow for direct visual comparison.

Pooled regional indicators for 2013–2022 are shown in Table 2. Pooled mean birth weight ranged from 3392.6 g (Almaty Region) to 3548.6 g (Mangystau Region); pooled mean birth length ranged from 52.2 cm (East Kazakhstan and Karaganda Regions) to 53.6 cm (Aktobe Region); the pooled Rohrer index ranged from 2.246 (North Kazakhstan Region) to 2.427 (Karaganda Region); pooled LBW ranged from 0.9% (Atyrau Region) to 5.3% (North Kazakhstan Region); and pooled macrosomia ranged from 11.7% (Almaty City) to 18.1% (Mangystau Region). Regions with a higher pooled LBW prevalence did not consistently overlap with regions with a higher pooled macrosomia prevalence (Figure 2), which is consistent with LBW and macrosomia warranting separate regional monitoring.

These pooled values are case-weighted (sum of cases 2013–2022 divided by sum of live births 2013–2022 within each region), which is equivalent, by construction, to how they were computed from the region-level row data; they are not an unweighted average of the 10 annual percentages, which for the same regions would give numerically different (though generally similar) values. Because regions could in principle differ in which years contributed more births, we additionally computed (i) a year-standardized version of Table 2, giving each of the 10 years equal weight regardless of its birth count, and (ii) a model-based estimate from a region + year fixed-effects model, averaged over years (Supplementary Table S11). All three approaches were numerically close for every region and outcome, indicating that annual-composition differences across regions are not a major driver of the pooled regional contrasts reported in Table 2. Notably, the officially registered live births varied in regions compared with the independent BNS national live-births (Supplementary Table S14).

### 3.3. National Trend-Model Estimates

Table 3 reports the national trend models for the three continuous outcomes, fitted on the 10 annual observations rather than the individual births, together with the standard error and exact *p*-value for each slope. Mean birth weight increased by 9.25 g/year (SE, 0.63; 95% CI, 7.79–10.71; *p* < 0.001; R^2^ = 0.964), corresponding to a model-estimated total increase of 83.3 g (95% CI, 70.1–96.4) over the study period. Mean birth length increased by 0.077 cm/year (SE, 0.006; 95% CI, 0.062–0.091; *p* < 0.001; total change of 0.69 cm, 95% CI of 0.56–0.82). The Rohrer index decreased by 0.0041/year (SE, 0.0004; 95% CI, −0.0051 to −0.0031; *p* < 0.001; total change of −0.037, 95% CI of −0.046 to −0.028). An autocorrelation-adjusted GLSAR(1) model gave a similar point estimate with an even wider interval (Supplementary Table S10), and it is reported as a sensitivity analysis.

Table 4 reports the national trend models for LBW and macrosomia, together with the standard error and exact *p*-value for the odds ratio and the annual absolute change in percentage points (with its 95% CI), in addition to the total change over the study period. Both outcomes showed substantial overdispersion under the standard binomial assumption (Pearson chi-square/df = 82.3 for LBW and 30.9 for macrosomia, both far above the value of 1 expected under simple binomial variance), so the quasi-binomial model is reported as primary. LBW decreased by an estimated OR of 0.947 per year (SE of the log-odds coefficient 0.011; 95% CI 0.927–0.967; *p* < 0.001). The model-estimated annual absolute change, evaluated at the mean study year (2017.5), was −0.133 percentage points/year (95% CI −0.185 to −0.082), and predicted prevalence was 3.16% in 2013 (95% CI 2.85–3.51) and 1.96% in 2022 (95% CI 1.74–2.20), a total model-estimated decrease of 1.21 percentage points over the decade. Macrosomia increased by an estimated OR of 1.033 per year (SE of the log-odds coefficient, 0.003; 95% CI, 1.027–1.039; *p* < 0.001). The annual absolute change was +0.398 percentage points/year (95% CI, 0.326–0.469), and predicted prevalence was 12.60% in 2013 (95% CI, 12.25–12.96) and 16.18% in 2022 (95% CI, 15.78–16.59), a total model-estimated increase of 3.58 percentage points.

### 3.4. Region-Specific Temporal Trends

A unified model containing region, year, and a region × year interaction term, fitted on the 170 region-year observations, showed a statistically significant region × year interaction for all five outcomes (birth weight F = 6.04, *p* < 0.001; birth length F = 13.60, *p* < 0.001; Rohrer index F = 17.64, *p* < 0.001; LBW F = 2.70, *p* < 0.001; macrosomia F = 8.44, *p* < 0.001; Table 5), confirming that the pace of change differed across territories rather than assuming this from the independent per-region regressions alone.

**Figure 3:**
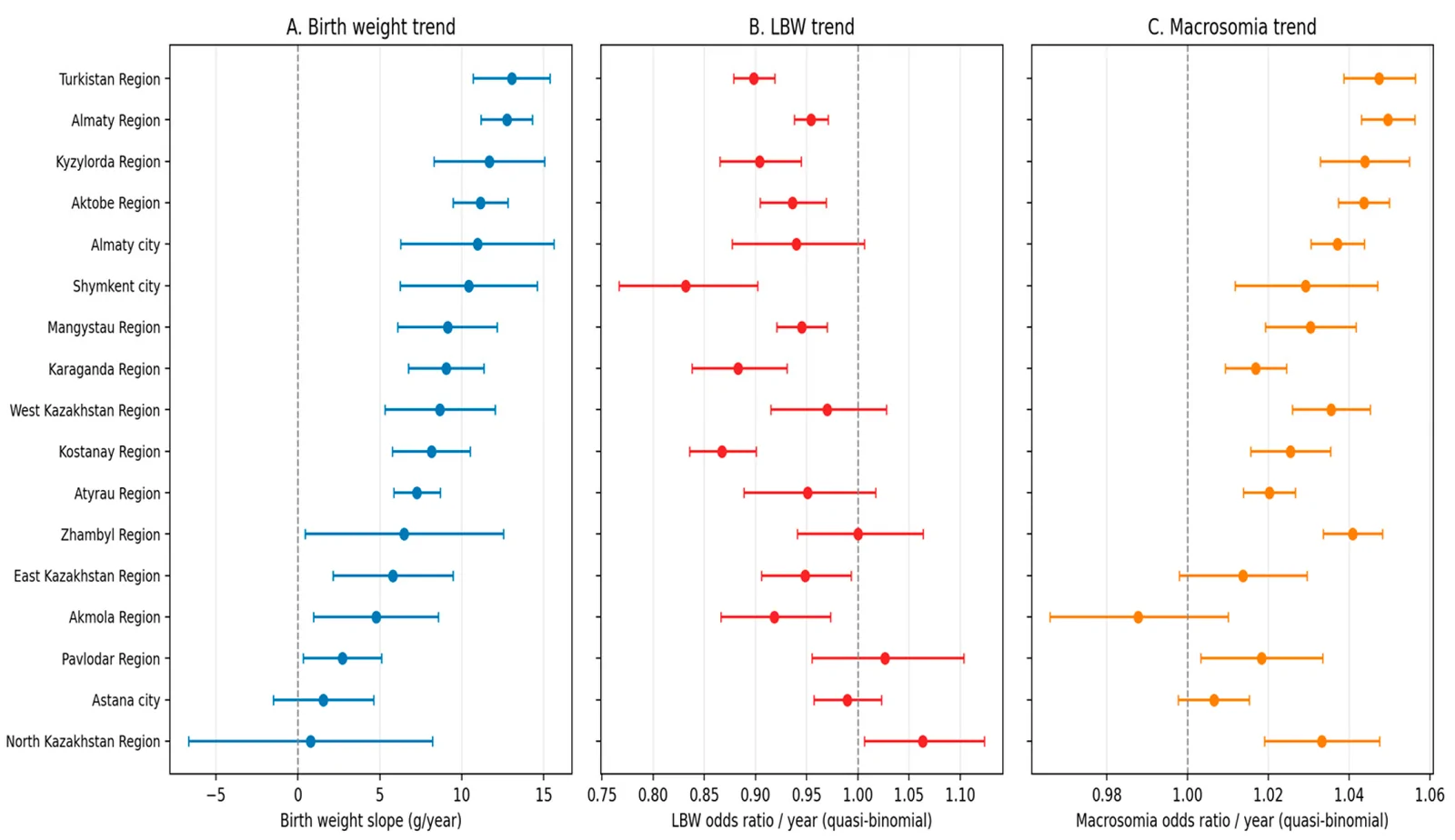
Region-specific annual trends with 95% confidence intervals, Kazakhstan, 2013–2022. (**A**) Birth-weight slope (g/year); (**B**) low-birth-weight odds ratio per year (quasi-binomial); and (**C**) macrosomia odds ratio per year (quasi-binomial). Regions are ordered by birth-weight slope. Dashed vertical lines mark no change (0 for panel (**A**); 1 for panels (**B**,**C**)).

Full region-specific estimates, both raw and false discovery rate (FDR)-adjusted across the 68 region-level tests performed (17 regions × 4 outcomes), are reported in Supplementary Tables S5 and S6. For birth weight, the largest annual increase was observed in Turkistan Region (12.7 g/year descriptively; FDR-adjusted *p* < 0.001), and the smallest, statistically non-significant change was observed in North Kazakhstan Region (0.8 g/year; FDR-adjusted *p* = 0.83). For birth length, the largest annual increment was observed in Shymkent City (0.21 cm/year, FDR-adjusted *p* < 0.001). After FDR correction, only North Kazakhstan Region retained a statistically significant upward LBW trend (OR 1.06/year, FDR-adjusted *p* = 0.040); the nominally significant upward trend previously reported for Pavlodar Region did not remain significant after correcting for overdispersion and multiple testing (FDR-adjusted *p* = 0.51). For macrosomia, FDR-adjusted trends were significantly upward in 14 of 17 regions; Akmola Region, East Kazakhstan Region, and Astana City did not show a statistically significant trend in either direction after correction (FDR-adjusted *p* = 0.32, 0.11, and 0.17, respectively), so the previously reported “modest decline” in Akmola Region is now reported as descriptive only. Figure 3 displays these region-specific estimates as a forest plot with 95% confidence intervals.

### 3.5. Sensitivity Analyses and Diagnostics

A linear-versus-quadratic F-test detected statistically significant curvature (*p* < 0.05) in the national annual series for birth weight, birth length, and the Rohrer index, but not for LBW or macrosomia (Supplementary Table S10); 2022 was the most influential single year (largest Cook’s distance) for birth weight, birth length, and the Rohrer index. In the pre-COVID (2013–2019) sensitivity analysis, slopes were systematically smaller than in the full 2013–2022 period (e.g., birth weight, +7.22 g/year; 95% CI, 5.25–9.18, versus +9.25 g/year for the full period), indicating that part of the full-period trend is driven by 2020–2022. Sex-stratified models showed similar direction and magnitude of trend for boys and girls for all five outcomes (Supplementary Table S10), so sex was not a major effect modifier of the national trends. Excluding East Kazakhstan Region, Almaty Region and Karaganda Region did not materially change the national birth-weight slope (8.9 g/year, 95% CI of 7.37–10.43, in the 14-region subset versus 9.25 g/year, 95% CI of 7.79–10.71, for all 17 regions) or the significance of the region × year interaction (*p* < 0.001), indicating the main regional conclusions are not driven by this simplification.

## 4. Discussion

### 4.1. Principal Findings

This nationwide ecological analysis of 3,483,810 live births in Kazakhstan, 2013–2022, found a statistically supported increase in mean birth weight and birth length, an observed decline in recorded LBW prevalence, and an increase in recorded macrosomia prevalence, together with statistically confirmed heterogeneity in regional trajectories. Because the source records lack gestational age or maternal characteristics, the observed changes are reported descriptively wherever a formal test was not available, and as statistically supported only where a corrected inferential model demonstrated it.

### 4.2. Interpretation of National Trends

The combination of increasing mean birth weight and length, declining recorded LBW, and rising macrosomia indicates that the national pattern should not be read as uniform improvement in perinatal health. Instead, it indicates a shift toward larger newborn size in which a decline in the proportion of newborns below 2500 g occurred alongside an increase in the proportion of very large newborns, consistent with prior evidence that the lower and upper tails of the birth-weight distribution can move in different directions over time [19,24]. The statistically estimated increase of 9.25 g/year in mean birth weight is small relative to the total variability in individual birth weight and should not be read as suggesting a clinically meaningful shift for an individual pregnancy. Therefore, its relevance is at the population level, where a shift of this size in the mean can nonetheless correspond to a detectable shift in the proportion of newborns crossing clinically meaningful absolute thresholds such as 2500 g or 4000 g, which is why we report LBW and macrosomia alongside the mean.

The decline in recorded LBW prevalence is directionally consistent with the international public-health priority of reducing the proportion of newborns weighing less than 2500 g. Recent global estimates indicate 19.8 million newborns (14.7% of live births) had LBW in 2020 [10]. However, the recorded national LBW prevalence of 1.46% in 2022 is unusually low relative to many international estimates: the same joint UNICEF-WHO modeling exercise that produced the global 14.7% figure [10] estimates Kazakhstan-specific LBW prevalence at approximately 5.4% in 2020, roughly three-and-a-half times the value recorded here for 2022. On the other hand, the two figures differ in reference year, as well as in data source and method—part of the roughly three-and-a-half-fold gap could, in principle, reflect two additional years of decline in Kazakhstan, since the recorded national LBW prevalence itself fell from 2.46% in 2020 to 1.46% in 2022, rather than the modeling-versus-registry contrast alone. This is a modeled estimate rather than a raw administrative count, so the two figures are not directly comparable on a like-for-like basis. Part of the gap may reflect the modeled estimate smoothing across data of variable quality across countries and years, while part may reflect genuine under-ascertainment or definitional differences in the Kazakhstan registry itself. We were not able to identify a Kazakhstan-specific, registry-based LBW estimate for direct comparison. Therefore, this cross-check against the modeled international estimate reinforces rather than resolves the caution we already place on the recorded 2022 value, and we do not treat either figure as a ground truth. LBW remains a composite indicator that cannot be interpreted mechanistically without gestational age and fetal growth classification [11,25,26]. A decline may reflect reductions in preterm birth, fetal growth restriction, changes in registration practice, or a combination, and this distinction also matters for term births, where LBW may reflect a specific combination of maternal, socioeconomic, and fetal-growth-related factors rather than prematurity alone [27]. Because the present dataset did not include gestational age, maternal age, parity, smoking, diabetes, body mass index, socioeconomic characteristics, or antenatal-care variables, the mechanisms behind the observed decline cannot be determined from these summary data alone and should be interpreted with caution.

The increase in recorded macrosomia prevalence is an important surveillance signal that warrants investigation alongside maternal metabolic and obstetric indicators, rather than a demonstrated consequence of any specific mechanism. The contemporary literature links fetal macrosomia and large-for-gestational-age birth with maternal obesity [28], diabetes [29], excessive gestational weight gain [30], multiparity, prolonged gestation, and male fetal sex. The International Diabetes Federation estimated that in 2024, 23.0 million live births (19.7%) were affected by some form of hyperglycemia in pregnancy [31]. We could not test whether the Kazakhstan trend reflects changes in maternal metabolic health, parity structure, gestational diabetes screening, obstetric practice, or demographic composition. Evidence from randomized trials indicates that structured antenatal diet and physical-activity interventions can reduce gestational weight gain; gestational diabetes; and some adverse neonatal outcomes, including large-for-gestational-age birth [32]. Whether such interventions would be relevant to the Kazakhstan trend is a question for future research linking registry data with maternal clinical records, not a conclusion supported by the present ecological data.

Although both birth weight and birth length increased, the mean Rohrer index declined. As described, the index was calculated for every individual newborn from that newborn’s own weight and length, and only then averaged within each year—it is the averaging step, not the calculation itself, that makes the reported national and regional figures group-level indicators. This decline in the mean should therefore not be interpreted as a shift in fetal growth patterns or individual newborn body proportionality. A population mean of individual indices describes how the index is distributed on average across the population in a given year or region, not how any particular newborn’s proportionality changed. Clinically, the Rohrer index evaluates individual proportionality, such as distinguishing symmetric from asymmetric growth restriction. In this population-level context, however, the downward shift merely indicates that birth length is increasing proportionally faster than the cube root of birth weight. Ultimately, this observation reflects the relative pace of two secular trends rather than physiological changes in individual newborns.

### 4.3. Regional Variations

Pooled regional estimates and observed trajectories varied across the harmonized territorial series. Western regions, including Mangystau, Atyrau, and West Kazakhstan, had among the highest pooled macrosomia proportions, whereas Almaty Region, Almaty City, and North Kazakhstan Region had lower pooled mean birth weight or higher pooled LBW. North Kazakhstan Region combined a comparatively low pooled mean birth weight, with the highest pooled LBW proportion and the only statistically significant (FDR-adjusted) upward LBW trend; Mangystau and Atyrau Regions, conversely, had low pooled LBW but high pooled macrosomia. This divergence supports monitoring the two ends of the birth-weight distribution separately by region, as well as nationally [24].

These regional patterns may reflect true differences in maternal health profiles, fertility patterns, access to antenatal and perinatal care, urban–rural composition, or socioeconomic conditions; they may equally reflect differences in registry completeness, referral practice (whether births are attributed to the mother’s region of residence or to the hospital’s location, which was not determinable from the source tables), or measurement and reporting practice across regions. Recent Kazakhstan-based evidence linking birth weight with infant mortality, and broader analyses of socioeconomic and demographic correlates of infant mortality, supports the need to interpret regional newborn indicators within a wider maternal, neonatal, and health-system context [22].

None of these possible explanations can be distinguished with the present non-adjusted data: the models are unadjusted for any covariate, as gestational age, maternal age, parity, diabetes, body mass index, and socioeconomic characteristics were not available in the source fields, and referral-related regional misclassification cannot be ruled out. Regions with a high pooled LBW proportion or a statistically supported upward LBW trend, and regions with high pooled macrosomia, therefore warrant validation using individual-level, gestational-age-specific, and data-quality-adjusted analyses before being used to target specific clinical interventions; the present study identifies which regions and which indicators warrant this closer look, but it does not explain the individual- or system-level mechanisms underlying the observed patterns.

### 4.4. Strengths and Limitations

Regarding novelty: To our knowledge, and based on the search of Kazakhstan-specific literature summarized in Section 1, this is the first analysis to jointly examine decade-long national and region-specific trends in birth weight, birth length, the Rohrer index, LBW, and macrosomia for Kazakhstan; we make this claim narrowly, with respect to the joint analysis of these five indicators together, rather than as a claim of novelty for any single indicator in isolation.

This analysis covers a full decade (3,483,810 live births) across all 17 harmonized regions of Kazakhstan, providing substantially broader national surveillance coverage than a single-center or cross-sectional study. Examining birth weight, birth length, the Rohrer index, LBW, and macrosomia together—rather than birth weight alone—provides joint information about average size, proportionality, and both tails of the birth-weight distribution; analyzing LBW and macrosomia together specifically demonstrated that trends at the lower and upper ends of the distribution moved in different directions, a pattern that an analysis focused only on LBW would have missed entirely. Combining national and regional analyses improved surveillance resolution: national data described the overall temporal pattern, while region-year data—tested formally via the unified interaction model, not asserted from pooled prevalence alone—revealed territorial variation concealed by the national average. Using a single official registry source across the full period may reduce heterogeneity from combining unrelated data sources, provided the source’s definitions and measurement units remained stable; however, we did not have documentation to confirm this directly and note it as an assumption rather than a demonstrated strength. The ecological design is appropriate for the stated surveillance objective: individual-level registry data are well suited to describing national and regional temporal patterns even though they cannot identify individual-level determinants [33], and this study’s explicit separation of population-level trends from individual-level risk is itself a methodological strength. The territorial harmonization procedure attempts to preserve comparability across administrative changes and is documented transparently, with a year-by-year mapping table (Supplementary Table S8) and a dedicated sensitivity analysis supporting it, rather than left as an undocumented judgment call. Finally, using a de-identified individual-level dataset improves transparency and reproducibility, which we have aimed to strengthen further by providing a data dictionary, the cleaned analytical dataset, and the complete analysis code deposited in a permanent public repository for replication.

This analysis has several important limitations. The analysis is unadjusted for individual-level covariates and describes population-level, not individual-level, associations; ecological fallacy is therefore possible, and individual-level risk factors cannot be inferred even though the underlying records are individual-level. The register covers inpatient deliveries only and is not a complete census of live births: comparison with independent BNS vital statistics shows 93.3% overall ascertainment, declining significantly from 95.1% in 2013 to 87.7% in 2022. Moreover, the source variables did not identify maternal place of residence or the reporting medical organization, so the mechanism used to assign records to territories could not be independently verified. Comparing regional record counts with independent BNS regional live-birth totals provides direct evidence that this matters: four regions have more inpatient records than their official resident birth count (coverage > 100%), most clearly Almaty City (127.8%) and Astana City (103.4%), consistent with referral of deliveries—particularly high-risk pregnancies—to tertiary hospitals in major cities rather than assignment by maternal residence. Almaty Region, immediately adjacent to Almaty City, shows one of the lowest regional coverage figures (81.9%), a pattern consistent with a donor–receiving relationship between the two. Karaganda and East Kazakhstan Regions also exceed 100% coverage, but these two regions, together with Almaty Region, are additionally affected by a separate data-consistency issue: the 2022 administrative split that created Abai, Zhetysu, and Ulytau Regions could not be fully reconciled between the two data sources for their parent regions (East Kazakhstan, Almaty Region, and Karaganda Region, respectively), so their individual coverage estimates should be interpreted with additional caution. Regional LBW and macrosomia estimates should therefore be interpreted as reflecting some unknown combination of true population differences, referral-driven regional misclassification, and residual ascertainment differences, rather than any one of these alone. Even with individual-level records, we could not check for duplicate records, implausible measurements beyond the exclusion thresholds applied, internal consistency between weight and length, or errors in regional/annual totals, because no further identifying or cross-linkage fields were available. The dataset does not report how many newborns were missing a valid birth weight or length measurement. If total live births were used as the denominator for LBW/macrosomia despite some missing anthropometric measurements, these prevalences may be under- or over-estimated relative to true population prevalence. We cannot determine whether weighing equipment, measurement protocols, rounding practices, staff training, or reporting procedures remained consistent across regions and years, and measurement heaping around 2500 g and 4000 g could directly affect LBW/macrosomia classification.

Without gestational age, the analysis cannot distinguish preterm LBW from term fetal growth restriction, or macrosomia from large-for-gestational-age birth, so the findings cannot be interpreted as direct evidence of improved or impaired fetal growth. Although annual sex proportions are reported and sex-stratified sensitivity models were fitted, the available aggregated data do not permit fully sex-standardized anthropometric analysis using sex-specific growth references. This study cannot account for changes in multiple births, maternal age, parity, ethnicity, migration, socioeconomic composition, diabetes, obesity, smoking, assisted reproduction, antenatal care, or mode of delivery, any of which may explain part of the observed temporal and regional variation—none of these fields are present in the source register at all, regardless of whether the records are aggregated or individual-level. Whether births were assigned to the mother’s region of residence or to the reporting hospital’s location could not be determined from the source fields. If hospital location was used, referral of high-risk pregnancies to tertiary centers could distort regional LBW/macrosomia estimates.

Administrative changes required regions to be merged, retained, or excluded across years. The harmonized regions may not correspond exactly to the administrative populations existing in every study year, and this may combine non-equivalent populations and reduce geographic precision, although the sensitivity analysis in Section 3.5 suggests the main regional conclusions are not driven by the three regions affected by the 2022 split. Despite the large number of births, the national trend analysis contains only 10 annual observations, thus limiting the assessment of non-linearity, structural breaks, temporal autocorrelation, and long-term cyclical variation. The large number of births does not compensate for the small number of annual time points, and if individual birth counts had been used to calculate standard errors for this year-level exposure, as in the original analysis, confidence intervals would have been excessively narrow because births within the same year share common reporting and healthcare-system conditions—a limitation we have addressed analytically but flag here as a general caution for interpreting any single-year comparison in this dataset. The models assume a constant linear trend across 2013–2022. The sensitivity analysis and diagnostics in Section 3.5 show this assumption is imperfect (statistically detectable curvature for three of five outcomes, and smaller slopes in the pre-COVID subperiod), so the estimated slopes may partly reflect the 2020–2022 period specifically, potentially related to the COVID-19 pandemic, changes in healthcare access, registration procedures, or the 2022 territorial reorganization.

The pooled 2013–2022 regional values may conceal within-region temporal change and should not be interpreted as current regional prevalence estimates (Section 3.2 reports year-standardized and model-adjusted alternatives that partly mitigate, but do not eliminate, this concern). The absolute thresholds used for LBW (<2500 g) and macrosomia (≥4000 g) are useful surveillance conventions but do not capture the full birth-weight distribution, and findings may differ under a 4500 g macrosomia threshold or under gestational-age- and sex-specific standards. A national or regional mean Rohrer index, even though calculated from individual-level values, still cannot describe individual body proportionality—that limitation follows from reporting a population mean, not from how the underlying index was calculated. Numerous region-specific comparisons were examined. We addressed this with FDR correction rather than leaving the regional findings uncorrected, but residual exploratory uncertainty remains given the number of outcomes and regions considered jointly. The annual totals and outcome prevalences, including the unusually low 2022 LBW prevalence, were compared against an internationally modeled Kazakhstan LBW estimate (Section 4.2) but not against an independent Kazakhstan-specific registry or vital-statistics LBW source, which we were unable to identify. This value in particular therefore still requires cautious interpretation pending a suitable in-country validation source. Finally, the series ends in 2022 and may not represent more recent patterns, and the findings are specific to Kazakhstan’s official reporting system and should not be generalized to other countries or interpreted as individual-level clinical risk estimates.

### 4.5. Implications for Surveillance and Future Research

These findings support continued national monitoring of LBW and indicate that macrosomia warrants more systematic attention in Kazakhstan’s maternal and child health surveillance, alongside external validation of the recorded LBW decline against an independent Kazakhstan-specific source if one becomes available. Future studies should link birth-registry data with maternal clinical records, gestational age, pregnancy complications, diabetes screening, maternal body mass index, gestational weight gain, delivery mode, and regional care indicators; only individual-level, linked data of this kind can distinguish whether the trends reported here reflect changes in fetal growth, maternal metabolic risk, registry coverage/completeness, regional care pathways, or broader demographic transitions. A structured comparison of urban and rural birth outcomes, and of regional economic indicators in relation to natality and birth-weight distribution, would also usefully extend this surveillance work but requires linkage to external data sources beyond the newborn registry used here.

## 5. Conclusions

Official registry data for Kazakhstan, 2013–2022, showed increases in recorded mean birth weight and length, an observed decline in recorded low-birth-weight prevalence, and an increase in macrosomia prevalence. Regional summary estimates and observed trajectories varied across the harmonized territorial series, with statistically confirmed heterogeneity in the pace of change. These findings support continued monitoring of both ends of the birth-weight distribution but do not identify the clinical, maternal, demographic, or data-registration mechanisms underlying the changes. Validation using individual-level records containing gestational age, maternal characteristics, and consistent regional coverage, together with comparison of the recorded 2022 low-birth-weight prevalence against an independent data source, is required before these trends can be interpreted as changes in fetal growth, maternal metabolic risk, or the quality of perinatal care.

## Figures and Tables

**Table 1:** Annual characteristics of the newborn population, Kazakhstan, 2013–2022.

Year	Total Live Births, n	Boys, n (%)	Birth Weight, Mean ± SD (g)	Birth Length, Mean ± SD (cm)	Rohrer Index, Mean ± SD	LBW, n (%)	Macrosomia, n (%)
2013	337,920	173,471 (51.3%)	3429.5 ± 498.3	52.5 ± 2.9	2.369 ± 0.311	10,454 (3.09%)	42,560 (12.59%)
2014	350,250	179,105 (51.1%)	3445.6 ± 500.3	52.6 ± 2.9	2.364 ± 0.315	10,299 (2.94%)	46,943 (13.40%)
2015	349,911	179,187 (51.2%)	3450.6 ± 495.4	52.7 ± 2.9	2.359 ± 0.303	9683 (2.77%)	46,783 (13.37%)
2016	351,062	180,774 (51.5%)	3457.9 ± 492.5	52.7 ± 2.8	2.359 ± 0.322	9202 (2.62%)	47,498 (13.53%)
2017	339,648	174,690 (51.4%)	3457.5 ± 499.3	52.7 ± 2.9	2.358 ± 0.302	9101 (2.68%)	46,591 (13.72%)
2018	345,988	177,638 (51.3%)	3472.0 ± 498.6	52.8 ± 2.8	2.358 ± 0.298	8798 (2.54%)	49,447 (14.29%)
2019	343,265	176,192 (51.3%)	3477.0 ± 497.2	52.9 ± 2.9	2.347 ± 0.307	8223 (2.40%)	49,928 (14.55%)
2020	362,712	186,546 (51.4%)	3493.8 ± 506.5	53.0 ± 2.9	2.342 ± 0.299	8930 (2.46%)	56,489 (15.57%)
2021	374,601	191,246 (51.1%)	3508.3 ± 501.2	53.1 ± 2.9	2.336 ± 0.285	8113 (2.17%)	60,782 (16.23%)
2022	328,453	167,586 (51.0%)	3518.4 ± 479.8	53.3 ± 2.7	2.328 ± 0.267	4797 (1.46%)	52,624 (16.02%)

LBW, low birth weight (<2500 g); macrosomia, birth weight ≥ 4000 g. For girls, n (%) is the arithmetic complement of the boys’ column shown here and is provided in full, by year and region, in Supplementary Table S12. Rohrer index calculated per individual newborn as (weight [g]/length [cm]^3^) × 100 before averaging (Section 2.5). Values use thousands separators throughout.

**Table 2:** Pooled regional anthropometric indicators, 2013–2022.

Region	Total Births, n	Birth Weight, Mean ± SD (g)	Birth Length, Mean ± SD (cm)	Rohrer Index, Mean	LBW, n (%)	Macrosomia, n (%)
Akmola Region	103,416	3473.6 ± 488.2	52.8 ± 2.7	2.358	2300 (2.2%)	13,494 (13.0%)
Aktobe Region	190,847	3489.4 ± 497.6	53.6 ± 2.8	2.268	4295 (2.3%)	29,044 (15.2%)
Almaty Region	288,090	3392.6 ± 520.2	52.5 ± 2.9	2.336	12,444 (4.3%)	34,141 (11.9%)
Almaty City	421,414	3411.4 ± 500.1	52.3 ± 2.8	2.381	17,084 (4.1%)	49,423 (11.7%)
Atyrau Region	152,431	3544.1 ± 479.7	52.9 ± 2.6	2.394	1441 (0.9%)	26,771 (17.6%)
East Kazakhstan Region	117,344	3454.9 ± 477.4	52.2 ± 2.4	2.419	2121 (1.8%)	15,279 (13.0%)
Zhambyl Region	251,781	3439.7 ± 524.8	53.0 ± 3.3	2.305	10,056 (4.0%)	34,356 (13.6%)
West Kazakhstan Region	120,021	3522.7 ± 490.7	52.7 ± 2.5	2.395	2025 (1.7%)	20,185 (16.8%)
Karaganda Region	194,839	3449.3 ± 486.5	52.2 ± 2.6	2.427	4542 (2.3%)	24,950 (12.8%)
Kostanay Region	100,327	3497.1 ± 474.1	53.4 ± 2.8	2.305	1393 (1.4%)	14,726 (14.7%)
Kyzylorda Region	172,923	3508.8 ± 492.8	52.6 ± 2.7	2.413	3090 (1.8%)	27,712 (16.0%)
Mangystau Region	182,767	3548.6 ± 488.2	53.4 ± 2.6	2.327	2404 (1.3%)	33,037 (18.1%)
Pavlodar Region	82,127	3496.5 ± 460.8	53.4 ± 2.6	2.297	847 (1.0%)	11,309 (13.8%)
North Kazakhstan Region	66,612	3396.4 ± 568.8	53.2 ± 3.3	2.246	3524 (5.3%)	8276 (12.4%)
Turkistan Region	555,302	3490.0 ± 482.0	52.7 ± 2.8	2.382	9835 (1.8%)	83,154 (15.0%)
Astana City	286,798	3486.9 ± 514.9	53.2 ± 3.0	2.318	8047 (2.8%)	44,331 (15.5%)
Shymkent City	196,771	3501.4 ± 466.3	53.5 ± 3.2	2.292	2152 (1.1%)	29,457 (15.0%)

Values are pooled 2013–2022 estimates, calculated as the sum of cases (or the sum of weight/length) divided by the sum of live births across all 10 years within each region (case-weighted); LBW, low birth weight (<2500 g); Rohrer index, mean of individual (weight/length^3^) × 100 values. Pooled SD is likewise computed directly from individual-level birth weight/length records combined across all 10 years within each region (i.e., from the combined sum and sum of squares of the individual values for that region, 2013–2022), not by averaging or otherwise combining the ten annual SDs shown in Table 1.

**Table 3:** National trend models, continuous outcomes.

Outcome	N	Slope/Year	SE	95% CI	*p*	Total Δ 2013–2022 (95% CI)	R^2^
Birth weight (g)	10	9.25	0.63	7.79, 10.71	<0.001	83.3 (70.1, 96.4)	0.964
Birth length (cm)	10	0.077	0.006	0.062, 0.091	<0.001	0.69 (0.56, 0.82)	0.951
Rohrer index	10	−0.0041	0.0004	−0.0051, −0.0031	<0.001	−0.037 (−0.046, −0.028)	0.914

Model: Outcome_mean ≈ β_0_ + β_1_·year_c, weighted least squares (weights = annual live-birth count), year_c = calendar year − 2013, N = 10 annual observations. SE and *p* refer to β_1_ (the annual slope). Total Δ = β_1_ × 9, with 95% CI from the corresponding linear combination of the model covariance matrix. See Supplementary Table S10 for observed-vs-fitted and residual diagnostics, the linear-vs-quadratic non-linearity test, the autocorrelation-adjusted (GLSAR) sensitivity model, and the 2013–2019 pre-COVID sensitivity analysis.

**Table 4:** National trend models, binary outcomes.

Outcome	N	OR/Year (95% CI)	SE (Log-Odds)	*p*	Annual Δ, pp (95% CI)	Total Δ, pp	Predicted 2013% (95% CI)	Predicted 2022% (95% CI)	Dispersion (df)	Residual Deviance (df)
Low birth weight (<2500 g)	10	0.947 (0.927, 0.967)	0.011	<0.001	−0.133 (−0.185, −0.082)	−1.21	3.16 (2.85, 3.51)	1.96 (1.74, 2.20)	82.3 (8)	691.9 (8)
Macrosomia (≥4000 g)	10	1.033 (1.027, 1.039)	0.003	<0.001	+0.398 (0.326, 0.469)	+3.58	12.60 (12.25, 12.96)	16.18 (15.78, 16.59)	30.9 (8)	247.0 (8)

Model: Cases/total live births ≈ logit^−1^(β_0_ + β_1_·year_c), quasi-binomial GLM (variance scaled by the estimated dispersion parameter), year_c = calendar year − 2013, and N = 10 annual observations (full model specification in Section 2.6.1). OR = odds ratio per one-year increase in calendar year; SE and *p* refer to β_1_ on the log-odds scale. Annual Δ = model-estimated annual absolute change in percentage points, evaluated at the mean study year (2017.5) via the delta method; Total Δ = predicted 2022 prevalence minus predicted 2013 prevalence. Dispersion = Pearson χ^2^/residual df; values far above 1 indicate overdispersion relative to the standard binomial assumption, motivating the quasi-binomial specification used here. pp = percentage point.

**Table 5:** Unified region × year interaction tests.

Outcome	F Statistic	Df	*p*-Value
Birth weight	6.04	16, 136	<0.001
Birth length	13.60	16, 136	<0.001
Rohrer index	17.64	16, 136	<0.001
Low birth weight	2.70	16, 136	<0.001
Macrosomia	8.44	16, 136	<0.001

## Data Availability

The data used in this study were obtained from the publicly available dataset “Data on newborns in the Republic of Kazakhstan without personal data”, published on the Open Data Portal of the Republic of Kazakhstan. The source of the dataset is the Electronic Register of Inpatients of the Ministry of Health of the Republic of Kazakhstan (ERSB MH RK). The dataset contained no direct identifiers, and the study team did not attempt re-identification. The de-identified individual-level analytical extract, the cleaned national-year and region-year aggregate datasets, the data dictionary, the territorial harmonization mapping, all supplementary tables, and the complete analysis code and methodology for data acquisition were deposited at https://doi.org/10.5281/zenodo.21617508.

## Supplementary Materials

The following supporting information can be downloaded at https://www.mdpi.com/article/10.3390/children13081069/s1, Table S1: Mean Birth Weight (g) by Region and Year, 2013–2022; Table S2: Mean Birth Length (cm) by Region and Year, 2013–2022; Table S3A: Proportion of Infants with Low Birth Weight (%) by Region and Year; Table S3B: Proportion of Infants with Macrosomia (%) by Region and Year; Table S4: Mean Rohrer Index by Region and Year, 2013–2022; Table S5: Region-Specific Trends in Birth Weight and Length, with FDR-Adjusted *p*-Values; Table S6: Region-Specific Trends in Low Birth Weight and Macrosomia (Quasi-Binomial), with FDR-Adjusted *p*-Values; Table S7: Data Dictionary; Table S8a: Raw (Pre-Harmonization) Region Labels Affected by Administrative Changes, by Year; Table S8b: Harmonized Regional Data Completeness, 2013–2022; Table S9: Record Exclusions by Criterion and Year; Table S10a: National Model Diagnostics: Non-Linearity and Influence; Table S10b: Autocorrelation-Adjusted (GLSAR) Sensitivity Models, National Continuous Outcomes; Table S10c: Pre-COVID (2013–2019) Sensitivity Analysis, National Outcomes; Table S10d: Sex-Stratified Sensitivity Analysis, National Outcomes; Table S11: Year-Standardized and Model-Adjusted Regional Estimates (vs. Case-Weighted Pooled, Table 2); Table S12: National and Regional Sex Distribution, 2013–2022; Table S13. Ascertainment of the Electronic Register of Inpatients relative to official annual national live-birth statistics, Kazakhstan, 2013–2022; Table S14. Ascertainment of the Electronic Register of Inpatients relative to official regional live-birth statistics, Kazakhstan, 2013–2022.

## Abbreviations

The following abbreviations are used in this manuscript:

AUC	Area under the curve
BMI	Body mass index
CI	Confidence interval
ERSB MH RK	Electronic Register of Inpatients of the Ministry of Health of the Republic of Kazakhstan
LBW	Low birth weight
OR	Odds ratio
SD	Standard deviation
