# Supplementary material for: Nationwide Trends in Newborn Anthropometric Indicators in Kazakhstan, 2013–2022: A Population-Level Time-Trend Analysis
Source: Children (Basel). 2026 Aug 12;13(8):1069. doi: 10.3390/children13081069 (PMC13511468; doi:10.3390/children13081069)
Supplement: Supplementary file 1 [file children-13-01069-s001.zip › children-4441343-supplementary.pdf]

Supplementary Materials

Table S1. Mean Birth Weight (g) by Region and Year, 2013–2022.

Region	2013	2014	2015	2016	2017	2018	2019	2020	2021	2022
Akmola Region	3464.3 ± 495.6	3450.1 ± 506.8	3447.5 ± 504.7	3483.1 ± 471.0	3454.5 ± 524.5	3470.6 ± 499.5	3501.8 ± 481.5	3490.2 ± 483.5	3499.4 ± 461.8	3478.4 ± 434.7
Aktobe Region	3438.8 ± 494.6	3458.1 ± 498.4	3463.9 ± 495.1	3468.8 ± 496.1	3477.4 ± 485.8	3485.4 ± 494.7	3499.0 ± 503.0	3514.3 ± 510.3	3528.7 ± 499.7	3549.1 ± 485.4
Almaty Region	3332.7 ± 528.4	3349.5 ± 513.8	3361.2 ± 510.5	3373.9 ± 514.7	3381.6 ± 512.8	3410.4 ± 510.5	3401.3 ± 525.9	3424.0 ± 529.4	3442.0 ± 536.9	3446.8 ± 503.7
Almaty city	3335.0 ± 523.6	3364.0 ± 523.1	3392.3 ± 493.9	3409.6 ± 485.6	3414.7 ± 485.4	3432.1 ± 476.4	3437.3 ± 468.5	3413.1 ± 539.3	3429.6 ± 529.6	3468.2 ± 455.3
Atyrau Region	3511.6 ± 470.9	3520.5 ± 481.9	3526.4 ± 478.0	3532.2 ± 477.4	3542.0 ± 477.9	3542.6 ± 480.5	3549.6 ± 470.0	3552.2 ± 495.1	3570.7 ± 487.5	3585.6 ± 469.9
East Kazakhstan Region	3443.8 ± 469.8	3442.9 ± 482.5	3443.3 ± 482.1	3446.1 ± 472.3	3439.1 ± 478.3	3451.8 ± 477.8	3440.8 ± 469.4	3473.1 ± 486.4	3499.9 ± 478.0	3499.9 ± 470.7
Zhambyl Region	3425.8 ± 487.7	3428.4 ± 512.8	3430.8 ± 505.1	3439.2 ± 499.7	3393.0 ± 573.7	3413.5 ± 572.6	3427.9 ± 552.0	3474.0 ± 514.2	3482.7 ± 509.4	3479.9 ± 508.3
West Kazakhstan Region	3488.6 ± 491.9	3503.0 ± 488.9	3491.4 ± 481.2	3489.0 ± 479.9	3522.7 ± 480.4	3534.1 ± 485.0	3532.6 ± 487.9	3559.5 ± 487.8	3535.1 ± 528.3	3570.4 ± 481.3
Karaganda Region	3405.8 ± 519.6	3425.8 ± 503.9	3430.9 ± 484.9	3445.8 ± 479.2	3450.6 ± 476.8	3455.4 ± 481.1	3450.2 ± 487.4	3462.2 ± 486.2	3493.1 ± 469.6	3501.6 ± 449.7
Kostanay Region	3459.4 ± 479.9	3473.8 ± 486.0	3465.4 ± 480.6	3484.3 ± 472.3	3507.7 ± 455.6	3510.8 ± 468.3	3522.7 ± 473.3	3515.2 ± 476.2	3528.4 ± 473.1	3523.3 ± 463.1
Kyzylorda Region	3462.2 ± 485.8	3472.0 ± 499.5	3493.9 ± 484.3	3486.7 ± 497.2	3486.9 ± 498.5	3496.2 ± 498.3	3510.4 ± 490.4	3533.5 ± 499.0	3557.1 ± 484.1	3578.3 ± 477.9
Mangystau Region	3485.5 ± 485.7	3521.5 ± 491.0	3535.4 ± 488.3	3536.5 ± 483.9	3542.0 ± 493.1	3570.6 ± 492.5	3547.1 ± 486.5	3574.2 ± 486.9	3575.1 ± 487.5	3584.3 ± 478.9
Pavlodar Region	3493.9 ± 454.4	3485.8 ± 463.0	3492.6 ± 465.0	3486.0 ± 457.9	3489.8 ± 447.5	3504.4 ± 443.5	3490.4 ± 441.7	3490.0 ± 477.8	3519.8 ± 477.7	3516.4 ± 474.3
North Kazakhstan Region	3423.2 ± 491.3	3432.9 ± 488.8	3367.7 ± 574.3	3351.1 ± 577.9	3375.7 ± 584.7	3391.9 ± 586.8	3389.4 ± 593.3	3404.1 ± 601.5	3412.3 ± 593.4	3424.8 ± 592.8
Turkistan Region	3433.5 ± 481.6	3456.7 ± 485.1	3465.0 ± 480.0	3469.2 ± 479.5	3469.0 ± 480.2	3490.9 ± 482.3	3505.9 ± 480.3	3531.5 ± 487.0	3548.2 ± 477.7	3550.3 ± 469.3
Astana city	3489.2 ± 510.9	3497.9 ± 501.0	3474.1 ± 520.3	3483.0 ± 518.8	3477.5 ± 517.0	3478.1 ± 517.6	3471.7 ± 530.2	3489.1 ± 517.2	3502.7 ± 511.1	3504.5 ± 501.7
Shymkent city	3441.4 ± 479.5	3478.5 ± 475.1	3481.5 ± 471.9	3488.1 ± 471.8	3472.2 ± 463.5	3473.0 ± 467.9	3492.8 ± 468.3	3529.2 ± 463.1	3541.7 ± 460.5	3543.8 ± 445.1

Values are mean ± SD birth weight (g) for all live births with a valid weight record in the given region-year cell..

Table S2. Mean Birth Length (cm) by Region and Year, 2013–2022.

Region	2013	2014	2015	2016	2017	2018	2019	2020	2021	2022
Akmola Region	52.8 ± 2.7	52.8 ± 2.9	52.7 ± 3.0	52.9 ± 2.7	52.7 ± 3.0	52.8 ± 2.8	52.8 ± 2.6	52.8 ± 2.6	53.0 ± 2.5	52.8 ± 2.5
Aktobe Region	53.3 ± 2.9	53.4 ± 2.9	53.4 ± 2.9	53.4 ± 2.8	53.5 ± 2.7	53.6 ± 2.8	53.7 ± 2.9	53.7 ± 2.9	53.8 ± 2.8	54.0 ± 2.7
Almaty Region	52.0 ± 2.9	52.2 ± 2.9	52.2 ± 2.8	52.3 ± 2.9	52.4 ± 2.9	52.6 ± 2.8	52.6 ± 2.9	52.8 ± 3.0	52.9 ± 3.0	53.1 ± 2.8
Almaty city	51.7 ± 3.0	51.9 ± 2.9	52.1 ± 2.7	52.1 ± 2.6	52.2 ± 2.6	52.4 ± 2.6	52.5 ± 2.5	52.3 ± 3.1	52.4 ± 3.0	52.8 ± 2.4
Atyrau Region	52.8 ± 2.7	52.8 ± 2.7	52.7 ± 2.5	52.7 ± 2.5	52.8 ± 2.5	52.7 ± 2.5	52.9 ± 2.5	53.2 ± 2.7	53.2 ± 2.6	53.1 ± 2.4
East Kazakhstan Region	52.3 ± 2.4	52.3 ± 2.4	52.2 ± 2.5	52.1 ± 2.5	52.0 ± 2.5	52.1 ± 2.5	52.0 ± 2.4	52.3 ± 2.5	52.5 ± 2.3	52.6 ± 2.3

Region	2013	2014	2015	2016	2017	2018	2019	2020	2021	2022
Zhambyl Region	52.9 ± 3.0	52.9 ± 3.3	52.9 ± 3.2	53.0 ± 3.2	52.7 ± 3.8	52.8 ± 3.7	53.0 ± 3.5	53.3 ± 3.1	53.4 ± 3.0	53.5 ± 3.0
West Kazakhstan Region	52.3 ± 2.5	52.6 ± 2.5	52.5 ± 2.4	52.6 ± 2.5	52.7 ± 2.4	52.7 ± 2.4	52.8 ± 2.5	53.1 ± 2.6	53.0 ± 2.8	53.1 ± 2.4
Karaganda Region	52.1 ± 2.9	52.2 ± 2.7	52.1 ± 2.5	52.3 ± 2.5	52.2 ± 2.5	52.0 ± 2.5	51.9 ± 2.5	52.0 ± 2.5	52.4 ± 2.5	52.3 ± 2.2
Kostanay Region	53.1 ± 2.8	53.2 ± 2.9	53.2 ± 2.9	53.2 ± 2.8	53.3 ± 2.7	53.5 ± 2.7	53.7 ± 2.8	53.5 ± 2.7	53.5 ± 2.6	53.6 ± 2.7
Kyzylorda Region	52.2 ± 2.7	52.3 ± 2.7	52.5 ± 2.6	52.5 ± 2.9	52.7 ± 2.8	52.6 ± 2.8	52.7 ± 2.7	52.8 ± 2.8	52.8 ± 2.6	52.9 ± 2.5
Mangystau Region	53.0 ± 2.6	53.3 ± 2.7	53.4 ± 2.6	53.4 ± 2.6	53.4 ± 2.6	53.4 ± 2.6	53.4 ± 2.6	53.4 ± 2.6	53.6 ± 2.6	53.6 ± 2.6
Pavlodar Region	53.4 ± 2.6	53.4 ± 2.6	53.4 ± 2.6	53.1 ± 2.5	53.3 ± 2.4	53.5 ± 2.4	53.6 ± 2.6	53.4 ± 2.8	53.4 ± 2.7	53.6 ± 2.7
North Kazakhstan Region	53.1 ± 2.7	53.2 ± 2.6	52.8 ± 3.3	52.8 ± 3.4	53.1 ± 3.4	53.3 ± 3.4	53.5 ± 3.5	53.5 ± 3.6	53.3 ± 3.4	53.3 ± 3.3
Turkistan Region	52.3 ± 2.9	52.5 ± 2.9	52.6 ± 2.9	52.7 ± 2.9	52.6 ± 2.8	52.7 ± 2.7	52.8 ± 2.7	52.9 ± 2.7	53.0 ± 2.6	53.1 ± 2.6
Astana city	53.0 ± 2.9	53.1 ± 2.9	53.1 ± 3.0	53.2 ± 3.0	53.1 ± 3.0	53.0 ± 3.0	53.1 ± 3.2	53.2 ± 3.0	53.3 ± 3.0	53.4 ± 2.9
Shymkent city	52.5 ± 3.0	52.8 ± 3.0	52.8 ± 2.9	52.9 ± 2.9	53.1 ± 3.1	53.3 ± 3.2	53.6 ± 3.2	53.9 ± 3.2	54.2 ± 3.3	54.3 ± 3.2

Values are mean ± SD birth length (cm).

Table S3A. Proportion of Infants with Low Birth Weight (%) by Region and Year.

Region	2013	2014	2015	2016	2017	2018	2019	2020	2021	2022
Akmola Region	2.41	2.96	2.99	1.88	3.28	2.63	1.53	1.83	1.41	1.08
Aktobe Region	2.90	2.77	2.57	2.40	2.17	2.09	2.39	2.32	1.92	1.03
Almaty Region	5.38	4.98	4.69	4.62	4.24	3.81	4.29	4.11	4.06	2.99
Almaty city	6.13	5.52	4.32	3.78	3.54	3.08	2.70	5.24	4.64	1.89
Atyrau Region	1.09	1.17	1.05	0.96	0.83	1.10	0.70	1.33	0.91	0.30
East Kazakhstan Region	1.66	2.07	2.42	1.80	2.09	1.87	1.75	1.78	1.16	0.86
Zhambyl Region	3.07	3.86	3.55	3.48	5.84	5.57	4.88	3.38	3.14	3.29
West Kazakhstan Region	2.18	1.87	1.87	1.71	1.34	1.50	1.50	1.24	2.46	1.10
Karaganda Region	3.99	3.23	2.57	2.13	1.86	2.15	2.46	2.18	1.27	0.64
Kostanay Region	2.17	1.96	2.06	1.85	0.98	1.02	1.01	1.01	0.83	0.53
Kyzylorda Region	2.44	2.53	1.81	2.18	2.00	1.98	1.69	1.72	1.01	0.64
Mangystau Region	1.76	1.39	1.47	1.33	1.52	1.40	1.39	0.98	1.15	0.87
Pavlodar Region	1.00	1.15	1.16	0.92	0.62	0.57	0.62	1.66	1.24	1.29
North Kazakhstan Region	2.96	2.55	5.70	6.51	5.69	5.99	6.28	6.19	5.90	5.48
Turkistan Region	2.47	2.35	2.17	2.03	1.96	1.77	1.44	1.40	1.01	0.81
Astana city	2.80	2.24	2.91	3.14	3.05	3.06	3.44	2.70	2.42	2.28
Shymkent city	2.09	1.73	1.53	1.54	1.53	1.66	1.16	0.56	0.48	0.10

LBW: birth weight <2500 g.

Table S3B. Proportion of Infants with Macrosomia (%) by Region and Year.

Region	2013	2014	2015	2016	2017	2018	2019	2020	2021	2022
Akmola Region	13.68	12.93	12.81	12.88	13.49	13.55	14.40	13.90	12.57	9.96
Aktobe Region	12.61	13.74	13.81	14.41	14.35	14.92	15.98	16.91	17.44	17.43
Almaty Region	9.68	10.20	10.52	10.96	11.30	12.27	12.48	13.03	14.27	13.68
Almaty city	9.53	10.45	10.81	11.34	11.53	11.95	12.03	12.86	13.16	12.91
Atyrau Region	15.97	16.94	17.27	17.07	17.34	17.19	17.52	17.80	18.95	19.27
East Kazakhstan Region	12.86	12.94	13.07	12.47	11.98	12.44	11.89	13.83	14.78	14.57
Zhambyl Region	11.66	12.54	12.34	12.87	12.69	13.70	13.98	15.28	15.60	15.80

Region	2013	2014	2015	2016	2017	2018	2019	2020	2021	2022
West Kazakhstan Region	14.95	15.92	15.00	14.98	16.61	17.31	17.13	18.51	18.38	19.24
Karaganda Region	11.99	12.91	11.83	12.40	12.87	12.85	12.96	13.23	13.87	13.77
Kostanay Region	13.11	13.96	13.48	14.07	15.02	15.04	15.93	15.06	16.58	15.28
Kyzylorda Region	13.92	14.43	15.10	14.62	14.84	15.37	16.02	17.45	18.54	19.36
Mangystau Region	14.85	17.13	17.43	17.32	17.59	19.39	17.85	19.00	19.79	19.69
Pavlodar Region	13.77	13.38	13.42	12.84	12.72	13.36	12.78	14.73	15.25	15.61
North Kazakhstan Region	11.74	11.68	11.45	10.55	11.67	12.86	13.21	13.78	13.80	14.42
Turkistan Region	12.55	13.64	13.73	13.68	14.06	15.00	15.54	16.96	17.89	17.56
Astana city	15.51	15.71	15.01	15.30	15.17	14.75	14.80	15.84	16.18	16.24
Shymkent city	12.88	14.55	14.35	14.80	13.14	13.59	14.50	16.20	17.07	15.92

Macrosomia: birth weight ≥4000 g.

Table S4. Mean Rohrer Index by Region and Year, 2013–2022.

Region	2013	2014	2015	2016	2017	2018	2019	2020	2021	2022
Akmola Region	2.35 ± 0.28	2.35 ± 0.41	2.36 ± 0.41	2.35 ± 0.39	2.36 ± 0.34	2.35 ± 0.29	2.38 ± 0.29	2.37 ± 0.38	2.35 ± 0.35	2.36 ± 0.27
Aktobe Region	2.28 ± 0.42	2.27 ± 0.29	2.27 ± 0.32	2.27 ± 0.30	2.28 ± 0.30	2.27 ± 0.34	2.26 ± 0.31	2.27 ± 0.33	2.27 ± 0.30	2.25 ± 0.25
Almaty Region	2.36 ± 0.29	2.35 ± 0.31	2.35 ± 0.29	2.35 ± 0.28	2.35 ± 0.31	2.34 ± 0.30	2.32 ± 0.29	2.32 ± 0.27	2.32 ± 0.29	2.30 ± 0.33
Almaty city	2.40 ± 0.29	2.39 ± 0.26	2.39 ± 0.27	2.40 ± 0.26	2.40 ± 0.26	2.38 ± 0.25	2.38 ± 0.23	2.37 ± 0.23	2.37 ± 0.24	2.34 ± 0.19
Atyrau Region	2.39 ± 0.31	2.40 ± 0.36	2.41 ± 0.26	2.42 ± 0.27	2.41 ± 0.25	2.42 ± 0.28	2.39 ± 0.26	2.36 ± 0.34	2.37 ± 0.29	2.39 ± 0.30
East Kazakhstan Region	2.40 ± 0.26	2.40 ± 0.30	2.42 ± 0.26	2.44 ± 0.30	2.44 ± 0.26	2.44 ± 0.28	2.44 ± 0.31	2.42 ± 0.29	2.42 ± 0.25	2.39 ± 0.21
Zhambyl Region	2.32 ± 0.32	2.32 ± 0.32	2.31 ± 0.32	2.31 ± 0.33	2.32 ± 0.33	2.31 ± 0.33	2.31 ± 0.36	2.29 ± 0.31	2.28 ± 0.29	2.27 ± 0.29
West Kazakhstan Region	2.43 ± 0.26	2.41 ± 0.25	2.41 ± 0.26	2.39 ± 0.25	2.40 ± 0.25	2.41 ± 0.32	2.39 ± 0.29	2.37 ± 0.29	2.37 ± 0.28	2.38 ± 0.21
Karaganda Region	2.40 ± 0.32	2.40 ± 0.30	2.42 ± 0.29	2.41 ± 0.28	2.42 ± 0.29	2.45 ± 0.27	2.46 ± 0.36	2.46 ± 0.27	2.43 ± 0.28	2.44 ± 0.25
Kostanay Region	2.32 ± 0.31	2.31 ± 0.40	2.30 ± 0.32	2.32 ± 0.32	2.32 ± 0.32	2.30 ± 0.32	2.28 ± 0.28	2.30 ± 0.39	2.30 ± 0.40	2.29 ± 0.28
Kyzylorda Region	2.43 ± 0.35	2.43 ± 0.35	2.42 ± 0.28	2.41 ± 0.39	2.38 ± 0.35	2.40 ± 0.31	2.40 ± 0.29	2.41 ± 0.29	2.42 ± 0.26	2.42 ± 0.26
Mangystau Region	2.33 ± 0.30	2.33 ± 0.29	2.31 ± 0.26	2.32 ± 0.24	2.32 ± 0.24	2.34 ± 0.26	2.33 ± 0.23	2.34 ± 0.24	2.32 ± 0.25	2.33 ± 0.25
Pavlodar Region	2.30 ± 0.27	2.29 ± 0.32	2.30 ± 0.33	2.33 ± 0.41	2.30 ± 0.25	2.29 ± 0.25	2.27 ± 0.33	2.29 ± 0.26	2.31 ± 0.21	2.29 ± 0.26
North Kazakhstan Region	2.29 ± 0.28	2.27 ± 0.30	2.27 ± 0.24	2.26 ± 0.26	2.24 ± 0.25	2.23 ± 0.27	2.20 ± 0.24	2.21 ± 0.25	2.23 ± 0.33	2.25 ± 0.26
Turkistan Region	2.40 ± 0.29	2.39 ± 0.30	2.38 ± 0.30	2.38 ± 0.40	2.38 ± 0.29	2.39 ± 0.28	2.38 ± 0.29	2.38 ± 0.26	2.37 ± 0.27	2.37 ± 0.25
Astana city	2.34 ± 0.35	2.34 ± 0.34	2.31 ± 0.35	2.31 ± 0.31	2.32 ± 0.34	2.33 ± 0.34	2.32 ± 0.37	2.31 ± 0.38	2.31 ± 0.32	2.30 ± 0.28

Region	2013	2014	2015	2016	2017	2018	2019	2020	2021	2022
Shymkent city	2.38 ± 0.27	2.36 ± 0.29	2.36 ± 0.27	2.36 ± 0.24	2.33 ± 0.37	2.30 ± 0.30	2.27 ± 0.33	2.26 ± 0.30	2.23 ± 0.24	2.22 ± 0.27

Rohrer index calculated per individual newborn as (weight [g] / length [cm]³) × 100, then averaged.

Table S5. Region-Specific Trends in Birth Weight and Length, with FDR-Adjusted p-Values.

Region	Weight β (g/year)	p (weight)	FDR-adjusted p (weight)	Length β (cm/year)	p (length)	FDR-adjusted p (length)
Akmola Region	4.77	0.0204	0.0289	0.012	0.3122	0.3424
Aktobe Region	11.14	<0.0001	<0.0001	0.073	<0.0001	<0.0001
Almaty Region	12.74	<0.0001	<0.0001	0.113	<0.0001	<0.0001
Almaty city	10.94	0.0006	0.0011	0.092	<0.0001	0.0002
Atyrau Region	7.26	<0.0001	<0.0001	0.056	0.0051	0.0080
East Kazakhstan Region	5.80	0.0064	0.0099	0.021	0.3408	0.3678
Zhambyl Region	6.48	0.0388	0.0507	0.063	0.0176	0.0255
West Kazakhstan Region	8.66	0.0003	0.0006	0.086	<0.0001	<0.0001
Karaganda Region	9.05	<0.0001	<0.0001	0.004	0.8009	0.8251
Kostanay Region	8.14	<0.0001	0.0001	0.061	0.0003	0.0005
Kyzylorda Region	11.67	<0.0001	0.0001	0.067	<0.0001	<0.0001
Mangystau Region	9.12	0.0001	0.0002	0.042	0.0031	0.0052
Pavlodar Region	2.71	0.0311	0.0414	0.021	0.1615	0.1894
North Kazakhstan Region	0.76	0.8198	0.8320	0.047	0.0574	0.0737
Turkistan Region	13.04	<0.0001	<0.0001	0.077	<0.0001	<0.0001
Astana city	1.56	0.2749	0.3169	0.034	0.0091	0.0138
Shymkent city	10.41	0.0004	0.0008	0.211	<0.0001	<0.0001

β estimated by weighted least squares of the 10 region-year means, weighted by region-year live-birth count. FDR = Benjamini-Hochberg false-discovery-rate correction applied jointly across all 68 region-level tests reported in Tables S5-S6. This 68-test family covers the four outcomes reported in Tables S5-S6 (birth weight, birth length, LBW and macrosomia); the Rohrer index was not included in this per-region multiplicity family because it is a deterministic function of birth weight and birth length, both already tested per region.

Table S6. Region-Specific Trends in Low Birth Weight and Macrosomia (Quasi-Binomial), with FDR-Adjusted p-Values.

Region	LBW OR/year	LBW dispersion	p (LBW)	FDR-adj. p (LBW)	Macrosomia OR/year	Macro. dispersion	p (Macro.)	FDR-adj. p (Macro.)
Akmola Region	0.918	15.6	0.0042	0.0067	0.988	12.4	0.2806	0.3180
Aktobe Region	0.936	10.6	0.0002	0.0003	1.044	1.9	<0.0001	<0.0001
Almaty Region	0.954	7.6	<0.0001	<0.0001	1.050	2.5	<0.0001	<0.0001
Almaty city	0.940	158.3	0.0748	0.0941	1.037	3.7	<0.0001	<0.0001
Atyrau Region	0.951	13.8	0.1442	0.1747	1.020	1.9	<0.0001	<0.0001
East Kazakhstan Region	0.949	8.9	0.0249	0.0345	1.014	6.9	0.0887	0.1096
Zhambyl Region	1.000	78.0	0.9957	0.9957	1.041	3.2	<0.0001	<0.0001
West Kazakhstan Region	0.970	14.3	0.3018	0.3364	1.036	3.0	<0.0001	<0.0001
Karaganda Region	0.883	22.7	<0.0001	<0.0001	1.017	2.5	<0.0001	<0.0001
Kostanay Region	0.867	3.7	<0.0001	<0.0001	1.026	2.5	<0.0001	<0.0001
Kyzylorda Region	0.904	12.3	<0.0001	<0.0001	1.044	5.5	<0.0001	<0.0001

Region	LBW OR/year	LBW dispersion	p (LBW)	FDR-adj. p (LBW)	Macrosomia OR/year	Macro. dispersion	p (Macro.)	FDR-adj. p (Macro.)
Mangystau Region	0.945	3.4	<0.0001	<0.0001	1.030	6.9	<0.0001	<0.0001
Pavlodar Region	1.026	9.5	0.4789	0.5089	1.018	4.7	0.0165	0.0245
North Kazakhstan Region	1.063	21.2	0.0292	0.0397	1.033	2.9	<0.0001	<0.0001
Turkistan Region	0.898	9.6	<0.0001	<0.0001	1.047	10.8	<0.0001	<0.0001
Astana city	0.989	18.0	0.5296	0.5540	1.007	5.9	0.1464	0.1747
Shymkent city	0.832	29.9	<0.0001	<0.0001	1.029	14.8	0.0010	0.0016

OR/year from a quasi-binomial GLM fitted within each region on its 10 region-year observations (point estimates identical to the original binomial model; standard errors and p-values scaled by the region-specific dispersion parameter, Pearson χ2/df). Dispersion values well above 1 indicate overdispersion relative to the standard binomial assumption. FDR = Benjamini-Hochberg correction jointly across all 68 region-level tests in Tables S5-S6 (four outcomes: birth weight, birth length, LBW and macrosomia). After this correction, several regions previously reported as showing a statistically significant LBW or macrosomia trend (e.g., Pavlodar Region for LBW; Akmola, East Kazakhstan and Astana city for macrosomia) are no longer statistically significant.

Table S7. Data Dictionary.

Original field (source)	English variable	Manuscript variable	Unit	Years available	Geographic level	Directly extracted or calculated
вес (weight)	weight	Birth weight	grams	2013–2022 (2023–2024 present but out of study window)	Individual record, aggregated to national-year and region-year	Directly extracted
рост (height)	height	Birth length	centimeters	2013–2022	Individual record, aggregated	Directly extracted
пол (gender)	gender	Sex	Мужской/Женский (male/female)	2013–2022	Individual record, aggregated	Directly extracted
дата рождения (birth_date)	birth_date	Birth date → calendar year	date (year derived)	2013–2022	Individual record, aggregated	Directly extracted; calendar year calculated from this field
регион (region_name)	region_name	Region (harmonized)	categorical, 17 levels after harmonization	2013–2022	Region	Directly extracted; harmonized per Table S8
— (derived)	lbw	Low birth weight indicator	binary (weight<2500g)	2013–2022	Individual record, aggregated	Calculated by the authors
— (derived)	macrosomia	Macrosomia indicator	binary (weight≥4000g)	2013–2022	Individual record, aggregated	Calculated by the authors

Original field (source)	English variable	Manuscript variable	Unit	Years available	Geographic level	Directly extracted or calculated
— (derived)	rohrer_index	Rohrer (ponderal) index	(g/cm³)×100, unitless ratio ×100	2013–2022	Individual record, aggregated	Calculated by the authors per individual newborn, then averaged (Section 2.5)

Source file: 'Data on newborns in the Republic of Kazakhstan without personal data' (Open Data Portal of the Republic of Kazakhstan, ashyq.data.gov.kz; original source system: Electronic Register of Inpatients, Ministry of Health of the Republic of Kazakhstan). Exact dataset URL/identifier, file version and download date are uploaded to https://doi.org/10.5281/zenodo.21617508.

Territorial harmonization rules applied (Section 2.3 of the main text): (1) records labelled 'г. Астана (действовавший до 23.03.2019)' were merged into 'Astana city'; the source file shows the pre-2019 label present in 2013–2018 (10,178–8,956 records/year) and absent from 2019 onward, coinciding with the March 2019 administrative boundary change, while the main 'Astana city' label is present in every year 2013–2022. (2) Records labelled 'Южно-Казахстанская область' (South Kazakhstan Region) were merged into 'Turkistan Region'; the source file shows this label present in 2013–2017 (2,213–1,011 records/year, tapering as Turkistan Region's own label took over) and absent from 2018 onward, coinciding with the 2018 renaming/split that also created Shymkent city as a separate administrative unit. Shymkent city is coded as a distinct category in the source file for all 10 study years and required no merging. (3) The three regions created in the 2022 territorial reorganization (Abai, Zhetysu, Ulytau) do not appear as separate categories anywhere in the 2013–2022 window of this data extract: records nominally attributable to Zhetysu and Ulytau appear only from 2023 onward (outside the study period), and no Abai-labelled record appears at all in the file made available to the analysis team. All three are therefore implicitly retained within their pre-2022 parent region throughout the study period (East Kazakhstan Region for Abai; Almaty Region for Zhetysu; Karaganda Region for Ulytau).

Table S8a. Raw (Pre-Harmonization) Region Labels Affected by Administrative Changes, by Year.

Source label	2013	2014	2015	2016	2017	2018	2019	2020	2021	2022
г. Астана (действовавший до 23.03.2019)	10,178	11,691	12,524	10,249	10,252	8,956	0	0	0	0
г. Астана	12,732	13,509	14,879	18,775	18,691	21,334	30,225	29,932	31,946	31,305
Южно-Казахстанская область	2,213	2,537	3,559	3,366	1,011	0	0	0	0	0
Туркестанская область	56,679	57,523	56,937	56,020	53,522	54,266	49,969	54,927	55,853	47,676
г. Шымкент	15,012	14,185	13,694	14,711	16,562	18,778	24,542	26,666	28,807	24,013

Raw record counts for the five source labels affected by the 2018-2019 administrative changes, prior to harmonization. All other 14 regions used a single consistent source label throughout 2013-2022.

Table S8b. Harmonized Regional Data Completeness, 2013–2022.

Harmonized region	Years with data	Completeness
Akmola Region	10/10	Complete
Aktobe Region	10/10	Complete
Almaty Region	10/10	Complete
Almaty city	10/10	Complete
Atyrau Region	10/10	Complete
East Kazakhstan Region	10/10	Complete
Zhambyl Region	10/10	Complete

Harmonized region	Years with data	Completeness
West Kazakhstan Region	10/10	Complete
Karaganda Region	10/10	Complete
Kostanay Region	10/10	Complete
Kyzylorda Region	10/10	Complete
Mangystau Region	10/10	Complete
Pavlodar Region	10/10	Complete
North Kazakhstan Region	10/10	Complete
Turkistan Region	10/10	Complete
Astana city	10/10	Complete
Shymkent city	10/10	Complete

All 17 harmonized regions have non-missing, non-zero data in all 10 study years after the merges described above; none required exclusion or interpolation for missing years. See Section 3.5 of the main text for the sensitivity analysis excluding the three regions (East Kazakhstan, Almaty oblast and Karaganda) affected by the 2022 split.

Table S9. Record Exclusions by Criterion and Year.

Year	Records in source file (2013–2022 window)	Excluded: weight out of range	Excluded: height out of range	Excluded: invalid/missing sex code	Final analytic n
2013	338,500	453	83	44	337,920
2014	350,609	218	86	55	350,250
2015	350,243	173	110	49	349,911
2016	351,395	206	92	35	351,062
2017	339,974	196	82	48	339,648
2018	346,316	177	125	26	345,988
2019	343,640	204	143	28	343,265
2020	363,133	232	176	13	362,712
2021	374,898	151	140	6	374,601
2022	328,698	102	134	9	328,453
Total	3,487,406	2,112	1,171	313	3,483,810

Sequential exclusion: weight out of range (<500g or >10,000g) applied first, then height out of range (<25cm or >70cm) among the remainder, then invalid sex code among the remainder, so percentages are not double-counted across criteria. Total records excluded across all criteria and years: 3,596 of 3,487,406 (0.10%). No values were imputed. Of the 2,112 weight-out-of-range exclusions, 2,099 records had a recorded birth weight <500 g and 13 had a recorded weight >10,000 g.

Table S10a. National Model Diagnostics: Non-Linearity and Influence.

Outcome	Linear R^2	Quadratic-term p-value	Max Cook's D (year)	Durbin-Watson
Birth weight (g)	0.9636	0.0440	0.392 (2022)	1.095
Birth length (cm)	0.9504	0.0163	0.682 (2022)	0.635
Rohrer index	0.9130	0.0118	0.705 (2022)	0.787
LBW (%)	0.8177	0.1321	1.831 (2022)	1.263
Macrosomia (%)	0.9307	0.1598	0.485 (2021)	1.386

Quadratic-term p-value from an F-test comparing a linear-in-year model against a quadratic-in-year model fitted to the 10 national annual observations. Durbin-Watson statistic computed on the linear model's residuals (values below ~1.5 indicate positive autocorrelation).

Table S10b. Autocorrelation-Adjusted (GLSAR) Sensitivity Models, National Continuous Outcomes.

Outcome	Slope/year	SE	95% CI	Estimated AR(1) ϱ
Birth weight (g)	9.7961	1.5218	6.1976, 13.3947	0.527
Birth length (cm)	0.1179	0.0364	0.0317, 0.2040	0.851

Outcome	Slope/year	SE	95% CI	Estimated AR(1) ρ
Rohrer index	-0.0069	0.0023	-0.0123, -0.0016	0.811

GLSAR(1) model estimates and adjusts for first-order autocorrelation between consecutive annual means; reported as a sensitivity analysis to the primary WLS model in Table 3 of the main text (Section 2.7).

Table S10c. Pre-COVID (2013–2019) Sensitivity Analysis, National Outcomes.

Outcome	Period	Slope or OR/year	95% CI
Birth weight (g)	2013-2022 (full)	9.2508	7.7869, 10.7147
Birth weight (g)	2013-2019 (pre-COVID)	7.2169	5.2500, 9.1838
Birth length (cm)	2013-2022 (full)	0.0766	0.0624, 0.0908
Birth length (cm)	2013-2019 (pre-COVID)	0.0571	0.0388, 0.0753
Rohrer index	2013-2022 (full)	-0.0041	-0.0051, -0.0031
Rohrer index	2013-2019 (pre-COVID)	-0.0028	-0.0042, -0.0013
LBW	2013-2022 (full)	OR=0.9466	0.9266, 0.9671
LBW	2013-2019 (pre-COVID)	OR=0.9606	0.9521, 0.9691
Macrosomia	2013-2022 (full)	OR=1.0330	1.0270, 1.0390
Macrosomia	2013-2019 (pre-COVID)	OR=1.0244	1.0177, 1.0312

Continuous outcomes: WLS slope/year. Binary outcomes: quasi-binomial OR/year. All slopes are systematically smaller in the pre-COVID subperiod, indicating the full-period trend is partly driven by 2020-2022 (Section 3.5 of the main text).

Table S10d. Sex-Stratified Sensitivity Analysis, National Outcomes.

Sex	Outcome	Slope or OR/year	95% CI
Girls	Birth weight (g)	9.3371	7.5430, 11.1312
Girls	Birth length (cm)	0.0755	0.0603, 0.0907
Girls	Rohrer index	-0.0038	-0.0048, -0.0028
Girls	LBW	OR=0.9454	0.9255, 0.9658
Girls	Macrosomia	OR=1.0364	1.0285, 1.0444
Boys	Birth weight (g)	9.2063	7.9246, 10.4881
Boys	Birth length (cm)	0.0779	0.0642, 0.0917
Boys	Rohrer index	-0.0044	-0.0055, -0.0033
Boys	LBW	OR=0.9479	0.9273, 0.9689
Boys	Macrosomia	OR=1.0312	1.0257, 1.0368

Sex-stratified national trend models (Section 2.7 of the main text). Direction and magnitude of trend are similar for boys and girls for all five outcomes.

Table S11. Year-Standardized and Model-Adjusted Regional Estimates (vs. Case-Weighted Pooled, Table 2).

Region	Weight, pooled (g)	Weight, year-std. (g)	Weight, model-adj. (g)	LBW, pooled (%)	LBW, year-std. (%)	LBW, model-adj. (%)	Macro., pooled (%)	Macro., year-std. (%)	Macro., model-adj. (%)
Akmola Region	3473.6	3474.0	3474.2	2.22	2.20	2.21	13.05	13.02	13.07
Aktobe Region	3489.4	3488.4	3488.5	2.25	2.26	2.26	15.22	15.16	15.17
Almaty Region	3392.6	3392.3	3392.4	4.32	4.32	4.31	11.85	11.84	11.84
Almaty city	3411.4	3409.6	3410.1	4.05	4.09	4.08	11.73	11.66	11.68
Atyrau Region	3544.1	3543.3	3543.0	0.95	0.94	0.95	17.56	17.53	17.50
East Kazakhstan Region	3454.9	3458.1	3460.1	1.81	1.75	1.75	13.02	13.08	13.23
Zhambyl Region	3439.7	3439.5	3439.9	3.99	4.01	3.98	13.65	13.65	13.64
West Kazakhstan Region	3522.7	3522.6	3522.4	1.69	1.68	1.69	16.82	16.80	16.79
Karaganda Region	3449.3	3452.1	3452.2	2.33	2.25	2.29	12.81	12.87	12.92

Region	Weight, pooled (g)	Weight, year-std. (g)	Weight, model-adj. (g)	LBW, pooled (%)	LBW, year-std. (%)	LBW, model-adj. (%)	Macro., pooled (%)	Macro., year-std. (%)	Macro., model-adj. (%)
Kostanay Region	3497.1	3499.1	3499.0	1.39	1.34	1.37	14.68	14.75	14.75
Kyzylorda Region	3508.8	3507.7	3508.0	1.79	1.80	1.79	16.03	15.96	15.98
Mangystau Region	3548.6	3547.2	3547.2	1.32	1.33	1.32	18.08	18.00	17.99
Pavlodar Region	3496.5	3496.9	3498.1	1.03	1.02	1.02	13.77	13.78	13.83
North Kazakhstan Region	3396.4	3397.3	3398.5	5.29	5.33	5.22	12.42	12.52	12.50
Turkistan Region	3490.0	3492.0	3491.5	1.77	1.74	1.75	14.97	15.06	15.03
Astana city	3486.9	3486.8	3484.7	2.81	2.80	2.84	15.46	15.45	15.36
Shymkent city	3501.4	3494.2	3494.9	1.09	1.24	1.13	14.97	14.70	14.67

Pooled = case-weighted (Table 2 of the main text, sum of cases/sum of births). Year-standardized = simple average of the 10 annual region-specific values, giving each year equal (1/10) weight regardless of its birth count. Model-adjusted = predicted region mean/prevalence from an outcome ~ region + year fixed-effects model, averaged over the 10 years. All three approaches are numerically close for every region, indicating annual-composition differences across regions are not a major driver of the pooled contrasts in Table 2 (Section 3.2 of the main text).

Table S12. National and Regional Sex Distribution, 2013–2022.

Region	Boys, n (%)	Girls, n (%)
Akmola Region	52,906 (51.2%)	50,510 (48.8%)
Aktobe Region	98,155 (51.4%)	92,692 (48.6%)
Almaty Region	147,712 (51.3%)	140,378 (48.7%)
Almaty city	216,978 (51.5%)	204,436 (48.5%)
Atyrau Region	78,445 (51.5%)	73,986 (48.5%)
East Kazakhstan Region	60,236 (51.3%)	57,108 (48.7%)
Zhambyl Region	129,538 (51.4%)	122,243 (48.6%)
West Kazakhstan Region	61,519 (51.3%)	58,502 (48.7%)
Karaganda Region	99,978 (51.3%)	94,861 (48.7%)
Kostanay Region	51,574 (51.4%)	48,753 (48.6%)
Kyzylorda Region	88,953 (51.4%)	83,970 (48.6%)
Mangystau Region	93,510 (51.2%)	89,257 (48.8%)
Pavlodar Region	41,013 (49.9%)	41,114 (50.1%)
North Kazakhstan Region	34,296 (51.5%)	32,316 (48.5%)
Turkistan Region	284,081 (51.2%)	271,221 (48.8%)
Astana city	147,013 (51.3%)	139,785 (48.7%)
Shymkent city	100,528 (51.1%)	96,243 (48.9%)

Pooled 2013-2022 sex distribution by region. National annual sex distribution by year is reported in Table 1 of the main text.

Supplementary Table S13. Ascertainment of the Electronic Register of Inpatients relative to official annual national live-birth statistics, Kazakhstan, 2013–2022.

Year	Eligible records, ERSB MH RK	Official live births, BNS	Not captured, n	Coverage, %
2013	337,920	355,315	17,395	95.1
2014	350,250	366,068	15,818	95.7
2015	349,911	366,636	16,725	95.4
2016	351,062	368,955	17,893	95.2
2017	339,648	359,271	19,623	94.5
2018	345,988	366,662	20,674	94.4
2019	343,265	370,567	27,302	92.6
2020	362,712	393,904	31,192	92.1

Year	Eligible records, ERSB MH RK	Official live births, BNS	Not captured, n	Coverage, %
2021	374,601	412,749	38,148	90.8
2022	328,453	374,363	45,910	87.7
Total 2013–2022	3,483,810	3,734,490	250,680	93.3

ERSB MH RK, Electronic Register of Inpatients of the Ministry of Health of the Republic of Kazakhstan (the analytical data source, covering inpatient deliveries only); BNS, Bureau of National Statistics of the Agency for Strategic Planning and Reforms of the Republic of Kazakhstan (independent official vital statistics). Abai region, Zhetysu Region and Ulytau Region were excluded, as they inflate the East Kazakhstan region, Almaty region and Karaganda Region and the comparisons become unreliable. Coverage is the eligible record count divided by the official live-birth total.

Supplementary Table S14. Ascertainment of the Electronic Register of Inpatients relative to official regional live-birth statistics, Kazakhstan, 2013–2022.

Region	Eligible records, ERSB MH RK	Official live births, BNS	Not captured, n	Coverage, %
Akmola Region	103,416	124,740	21,324	82.9
Aktobe Region	190,847	200,102	9,255	95.4
Almaty Region	288,090	351,675	63,585	81.9
Almaty city	421,414	329,808	-91,606	127.8
Atyrau Region	152,431	169,654	17,223	89.8
East Kazakhstan Region	117,344	107,592	-9,752	109.1
Zhambyl Region	251,781	281,977	30,196	89.3
West Kazakhstan Region	120,021	131,425	11,404	91.3
Karaganda Region	194,839	188,195	-6,644	103.5
Kostanay Region	100,327	118,865	18,538	84.4
Kyzylorda Region	172,923	202,719	29,796	85.3
Mangystau Region	182,767	202,902	20,135	90.1
Pavlodar Region	82,127	121,060	38,933	67.8
North Kazakhstan Region	66,612	73,303	6,691	90.9
Turkistan Region	555,302	577,880	22,578	96.1
Astana city	286,798	277,400	-9,398	103.4
Shymkent city	196,771	275,193	78,422	71.5

ERSB MH RK, Electronic Register of Inpatients of the Ministry of Health of the Republic of Kazakhstan (the analytical data source, covering inpatient deliveries only); BNS, Bureau of National Statistics of the Agency for Strategic Planning and Reforms of the Republic of Kazakhstan (independent official vital statistics). Abai region, Zhetysu Region and Ulytau Region were excluded, as they inflate the East Kazakhstan region, Almaty region and Karaganda Region and the comparisons become unreliable. Coverage is the eligible record count divided by the official live-birth total.
